# Structural and Plasmonic Evolution in Mixed-Dimensionality Bismuth/Graphene Heterostructures

**DOI:** 10.1021/acsami.5c20752

**Published:** 2026-03-03

**Authors:** Tushar Gupta, Kenan Elibol, Michael Stöger-Pollach, Kimmo Mustonen, Clemens Mangler, Jannik C. Meyer, Jani Kotakoski, Bernhard C. Bayer, Dominik Eder

**Affiliations:** † Institute of Materials Chemistry, 27259 Technische Universität Wien (TU Wien), Getreidemarkt 9/165, A-1060 Vienna, Austria; ‡ University of Vienna, Faculty of Physics, Boltzmanngasse 5, A-1090 Vienna, Austria; § 28326 Max Planck Institute for Solid State Research, Heisenbergstrasse 1, 70569 Stuttgart, Germany; ∥ USTEM, Technische Universität Wien (TU Wien), Wiedner Hauptstrasse 8-10, A-1040 Vienna, Austria; ⊥ Institute of Applied Physics, 27258 Eberhard Karls University of Tuebingen, Auf der Morgenstelle 10, D-72076 Tuebingen, Germany

**Keywords:** bismuth, graphene, *in situ* TEM, physical vapor deposition, crystallization, mixed-dimensionality heterostructures, electron beam induced effects, electron energy loss spectroscopy

## Abstract

Mixed-dimensionality heterostructures of low-dimensional bismuth (Bi) with two-dimensional (2D) graphene are of interest in a variety of application fields ranging from nanoelectronics, next-generation batteries, and (photo)­catalysis to plasmonics. We here explore the evolution of the morphology and structure of low-dimensional Bi/graphene heterostructures by high-resolution (scanning) transmission electron microscopy ((S)­TEM). To this end, we deposit low-dimensional Bi nanostructures onto suspended monolayer graphene membranes via physical vapor deposition (PVD). This enables us to study intrinsic Bi–graphene interactions, in contrast to prior work that utilized Bi on supported graphene. We find that Bi deposited onto room temperature graphene consists of grains formed by irregularly shaped β-Bi crystals with a β-Bi[001]⊥graphene(001) texture and β-Bi nanorods with a β-Bi[2–21]⊥graphene(001) texture. Importantly, both texture types show rotational van der Waals epitaxy with the supporting graphene. The room temperature depositions grow via an initial amorphous β-Bi[2–21]-like state into a closed film of β-Bi structure. For higher graphene temperatures of 150 to 250 °C during deposition, we find the formation of amorphous Bi nanoparticles (NPs) at much reduced coverage due to Bi reverse desorption at these temperatures. While the room temperature deposited Bi films remain static under the electron beam in (S)­TEM, the amorphous Bi NPs from higher temperature depositions exhibit electron beam induced *in situ* crystallization in TEM. In parallel to observing their structural evolution during this crystallization, this also enables us to probe the evolution of plasmonic features of Bi NPs via (valence) electron energy loss spectroscopy ((V)­EELS), suggesting a link between crystallization state and Bi NP surface plasmon (SP) energy.

## Graphical abstract

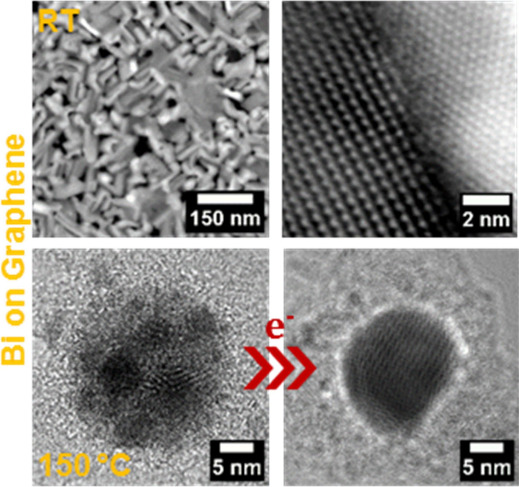

## Introduction

The allotropic wealth of low-dimensional pnictogens including antimony (Sb) and bismuth (Bi) has recently garnered tremendous attention.^1^ This interest also includes mixed-dimensionality heterostructure formation,^2,3^ in which a low-dimensional (zero-, one-, or two-dimensional (0D, 1D, or 2D)) pnictogen is grown atop of a 2D material like graphene.^4^

We here focus on the heterostructure formation of low-dimensional Bi including nanoparticles (NPs) on 2D graphene. Low-dimensional Bi is of high current interest due to its exotic electronic properties including readily emerging quantum size effects,^5^ topological insulation,^6^ and large thermoelectric power;^7^ its high suitability as an anode material in next-generation batteries;^8^ its strong coupling with light as a plasmonic material;^9,10^ and its catalytic activity as a potential material for (photo)­catalysis^11^ and possibly even plasmonic catalysis.^12^

All these Bi applications are intimately linked to the structure of the low-dimensional Bi, which is also intimately linked to the nature of the support of the Bi, making heterostructure formation key in Bi applications. Among the various Bi heterostructure supports, graphene has been found to be of particular importance for various applications: In 2D electronics, integration of 2D Bi (“bismuthene”) with graphene has been found to eliminate metal induced gap states, thereby reducing Fermi-level pinning and enhancing the effective channel length because of weak van der Waals (vdW) interaction and thus mitigating contact resistances.^13^ Also, Bi/graphene has been found to be useful in electrochemical sensing^14^ applications. In energy applications, Bi/graphene heterostructures were found to be a promising electrode material for next-generation batteries.^15^ In catalysis, mixed-dimensionality Bi/graphene heterostructures have recently proven their photocatalytic activity under ultraviolet to infrared excitation with long-term performance stability.^16^

Another key application field for Bi NPs is plasmonics,^10^ including the demonstration of switchable localized optical resonances, paving the way toward possible applications in optical switches.^9^ Key to useful optical switching are plasmonic materials that are (i) responsive to external stimuli and (ii) support optical resonances in the UV.^9^ In particular, plasmonic nanostructures that exhibit phase transitions are found to be suitable candidates for such switching applications.^17^ Compared to conventional noble metal plasmonic structures,^9^ Bi NPs can have external stimuli dependent phase transitions above room temperature, including readily accessible melting/solidification,^17^ and show a contrast in dielectric function from the UV to near IR range on account of the melting and solidification processes.^18^ Ultrathin graphene may be a useful support material for such plasmonic applications of nanostructured Bi.^19^ Also in this context, a further possible confluence of the plasmonic and photocatalysis applications of Bi on graphene is plasmonic catalysis, which is currently an emerging concept.^12^

In general, to date, the exact structure relations in mixed-dimensionality Bi/graphene heterostructures often remain underexplored, particularly under dynamic conditions and at high resolution. Prior work has investigated mixed-dimensionality Bi/graphene heterostructures but largely relied on ultrahigh-vacuum (UHV) scanning tunneling microscopy (STM) and also always had additional support which, depending on the strength of graphene/support interactions, can obscure the intrinsic Bi/graphene interactions.^20–30^

In contrast, we here study heterostructures formed from low-dimensional Bi via scalable physical vapor deposition (PVD) on truly freestanding monolayer graphene membranes. Hereby, the graphene membranes act not only as a 2D component in our heterostructures but also as an ideal substrate to make the low-dimensional Bi structure readily accessible to investigation by atomic-resolution (scanning) transmission electron microscopy ((S)­TEM), due to the low scattering background of graphene in (S)­TEM.^4,31,32^ We thereby first map out a parameter space of low-dimensional Bi morphology and structure on freestanding graphene down to atomic resolution as a function of archetypal Bi PVD conditions. This also includes investigating van der Waals (vdW) epitaxial effects between the Bi and the freestanding graphene in these heterostructures. Then, using the energy input of the electron beam in (S)­TEM as a proxy for *in situ* annealing,^31,32^ we probe the structural evolution of Bi/graphene heterostructures. Notably, we concurrently also follow the plasmonic properties of the Bi NPs as a function of their structural evolution by time-resolved *in situ* (valence) electron energy loss spectroscopy ((V)­EELS).

## Experimental Methods

Nominal thicknesses of 2 to 20 nm Bi were deposited via PVD (thermal evaporation of Bi powder, 99.999% purity, average particle size of 150 μm, Goodfellow, in a MANTIS deposition system QUBE series) onto suspended, chemical vapor deposited^33^ (CVD) monolayer graphene membranes (commercial Graphenea CVD monolayer graphene-covered Quantifoil TEM grids). The nominally deposited Bi thickness was measured *in situ* by a nonheated, concurrently exposed quartz crystal microbalance (QMB). The base pressure of the PVD system was ∼10^–5^ mbar. Deposition was carried out at substrate temperatures of nominally room temperature (RT), 150 °C, and 250 °C, whereby sample heating and leaving them to cool naturally were done under vacuum. The produced heterostructures were then examined via (S)­TEM after transport and storage in ambient air. TEM employed a FEI TECNAI F20 FEG TEM operating at 60 kV, acquiring bright-field (BF) and selected area electron diffraction (SAED) data. STEM employed a Nion UltraSTEM100 operating at 60 kV, acquiring high-angle annular dark-field (HAADF, 80–200 mrad) data.^4,31,32^ Additionally, Raman spectroscopy was measured with a Horiba Jobin-Yvon LabRAM 800HR spectrometer (532 nm).^34^ Note that Raman spectra were measured on Bi that had been deposited onto CVD graphene remaining on its Cu foil growth catalyst.^4,33^ This was necessary in order to obtain Raman spectra without degradation of the freestanding Bi/graphene heterostructures from incident laser irradiation. Figure S1 confirms the high quality of the monolayer graphene that has been used as the substrate for Bi depositions both in suspended CVD film form and as a CVD film still on the Cu foil growth catalyst support. These measurements employed a WITec alpha 300 RSA+ system with a laser wavelength of 488 nm, which reduces the photoluminescence background from the Cu support compared to 532 nm excitation.^34^

Please note that in this report, when describing the β-Bi phase,^35^ we employ for labeling of the β-Bi (*hkl*) planes and [*uvw*] directions predominantly the following structure files: β-Bi (A7, rhombohedral, *R*3̅*m*): 04-007-5315/53796 (from the PDF4+ crystallographic database/Inorganic Crystal Structure Database ICSD collection code) and for graphene (0-056-0159). Figure S2 shows salient simulated electron diffraction (ED) patterns of β-Bi along the [001] (β-Bi(001)) and [2–21] zone axes and the graphene [001] zone axis (graphene(001)), along with their atomic models in both top and side views.

Phase analysis of (S)­TEM data employed primarily ED pattern simulation using Highscore Plus/PDF4+ software (ICDD PDF4+ 2020 RDB, software version 4.20.0.1, database version 4.2001) for manual matching of measured SAED and simulated ED patterns. Structure visualization was done by Vesta software.

We want to underscore that we use freestanding CVD graphene films, which show a typical “patchwork quilt” grain structure with monolayered grains of several micrometer lateral grain sizes stitched together in a monolayer graphene film with rotational offset between adjacent monolayer grains.^36^ Therefore, often employed integral techniques such as X-ray diffractometry (XRD) with typical lateral regions of interest in the millimeter range would therefore average over many multiple graphene grains with different rotational orientations, thus likely leading to smeared out and hard to interpret information on any possible epitaxial Bi/graphene relations from such XRD measurements. Also, such XRD measurements would likely require the graphene to remain on a solid support (as no monolayer graphene films can, to date, be suspended over more than a few micrometer sizes without additional support grids),^37^ while we here aim to probe the Bi/graphene interactions deliberately without a substrate under the graphene. This motivates our use of (S)­TEM and SAED as the techniques of choice in this study.

## Results and Discussion

### Bi PVD Parameter Space

We first compare in Figure 1a–c the Bi morphology as a function of controlled graphene membrane substrate deposition temperature (RT, 150 °C, and 250 °C) for a fixed nominal Bi thickness of 10 nm. The RT deposition yields almost fully covering film-like deposits composed of predominantly pronounced, faceted grains (Figures 1a, 2, and S3, areal coverage of ∼95%). For the higher temperature depositions at 150 and 250 °C, the deposits change to small (projected diameter range from ∼3 to 20 nm), isolated, nonfaceted NPs (Figures 1b,c and S4). Concurrently, the amount of deposited Bi drastically decreased with increasing substrate temperature (to an areal coverage of ∼3% for 150 °C and ∼0.4% for 250 °C, see Figure S3). We ascribe this reduction of the amount of Bi deposition with increasing substrate temperature to greatly increased backward Bi desorption during deposition at higher substrate temperatures.^4,38^

**Figure 1:**
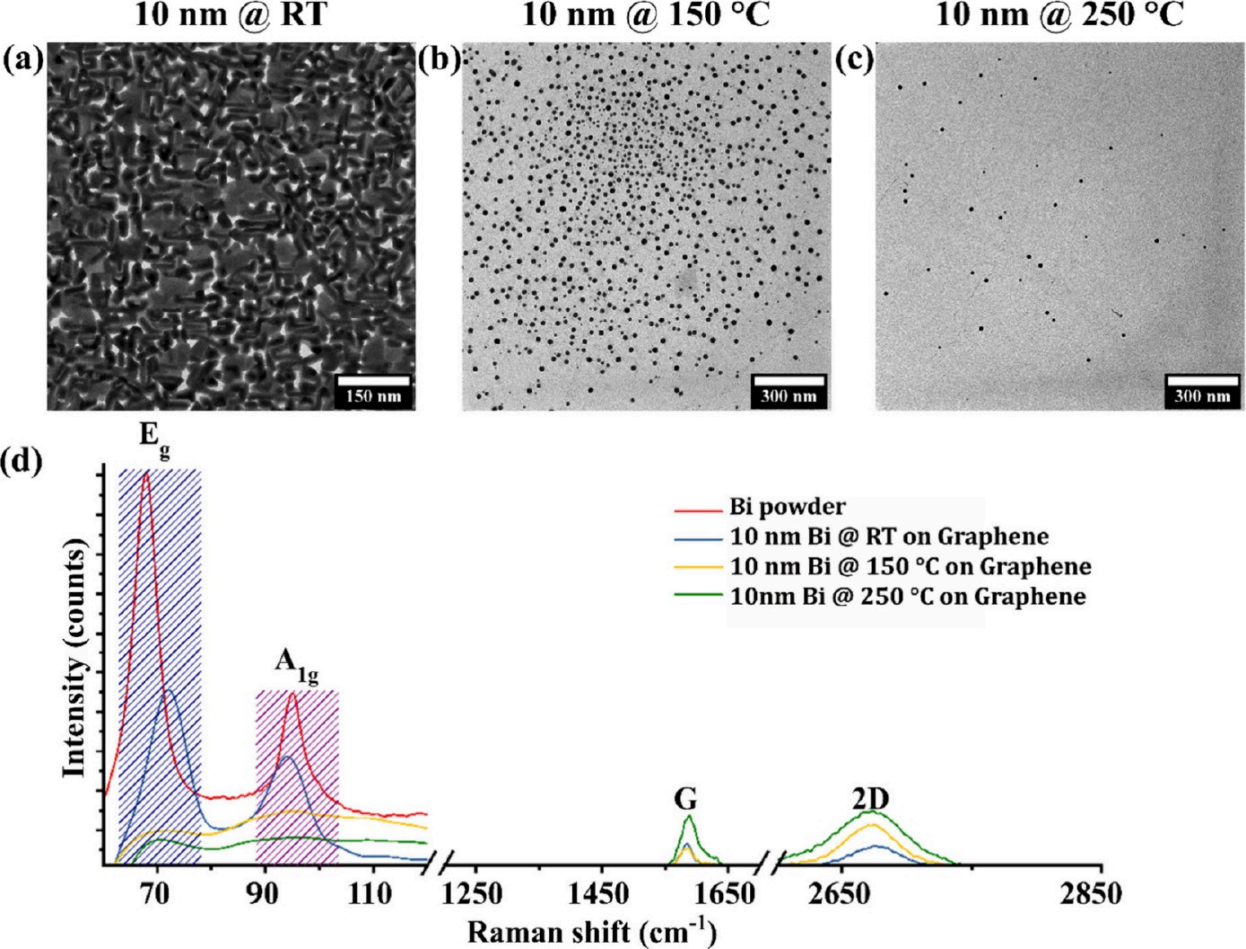
Bi deposition. BF-TEM micrographs of nominally 10 nm Bi on freestanding CVD monolayer graphene membranes deposited at (a) RT, (b) 150 °C, and (c) 250 °C substrate temperatures. (d) Raman spectra measured on nominally 10 nm Bi on CVD graphene (remaining on Cu CVD catalyst foil) deposited at RT, 150 °C, and 250 °C and additionally, for reference, bulk Bi powder. E_g_ and A_1g_ Raman peaks are labeled for Bi,^41,42^ as well as the G and 2D peaks for graphene, while no appreciable graphene defect-related D peak is observed.^33^ The Raman data in (d) were measured directly on the CVD graphene films remaining on their Cu growth support catalyst foils when using a Raman excitation laser with a 532 nm wavelength (green). The use of this green laser wavelength and this particular setup was due to technical availability reasons for ensuring a low enough edge filter position to also obtain sufficient signal at the low wavenumbers relevant for Bi signals. The use of a green laser for measurements of graphene on Cu is, however, not ideal since it induces a strong Cu-related photoluminescence background, as well as peak broadening and intensity ratio changes.^34^ For the 1250 to 2850 cm^–1^ graphene-related region in (d), the strong Cu support-related photoluminescence background from use of a 532 nm green laser^34^ was manually removed. To therefore further confirm the monolayered and high-quality nature of the graphene layer used for Bi deposition, we include in Figure S1 Raman spectra measured with a 488 nm (blue) laser, which greatly reduces the Cu-related photoluminescence and associated peak intensity changes and peak broadening.^34^ The Raman data in Figure S1 clearly confirm that our graphene layers are monolayered (via the 2D/G intensity ratio, consistent with high-quality CVD graphene)^33,34^ and of high quality (low D signal, consistent with high-quality CVD graphene).^33,34^

**Figure 2:**
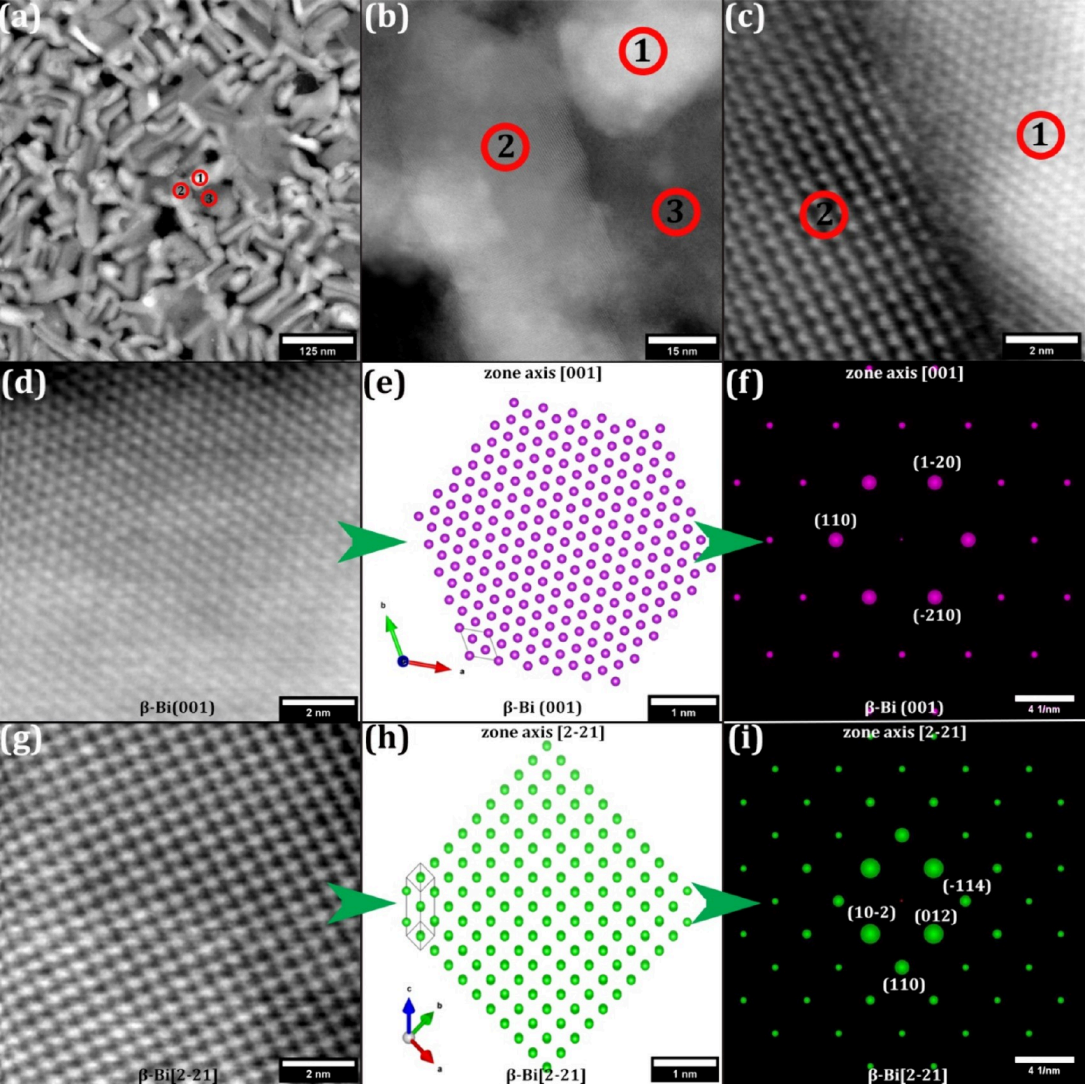
RT Bi depositions (texture). HAADF STEM images of 10 nm Bi films on graphene at RT, outlining three different regions in an overview (a, b) and at atomic resolution (c). (d–f) Atomically resolved STEM image (d), atomic model (as in ADF-STEM) (e), and corresponding simulated ED pattern (f) of β-Bi(001), marked as region 1 in (a–c). The simulated ED pattern is indexed to β-Bi(001), i.e., β-Bi viewed along the [001] zone axis. (g–i) Atomically resolved STEM image (g), atomic model (as in ADF-STEM) (h), and corresponding simulated ED pattern (i) of β-Bi[2–21], marked as region 2 in (a–c). The simulated ED pattern is indexed to β-Bi viewed along the [2–21] zone axis. For further information on atomic models and ED simulations, see Figure S2. For the atomically resolved STEM image and FT pattern of region 3, see Figure S5. The corresponding unit cells and zone axes for the atomic models have been indicated.

Figure 1d shows the Raman spectra of the as deposited Bi depositions on graphene from RT, 150 °C, and 250 °C depositions against the reference Raman spectrum of crystalline Bi bulk powder.^39,40^ For bulk Bi powder, we measure two signature first order E_g_ and A_1g_ Raman^41^ modes of the β-Bi structure at 68 and 95 cm^–1^, respectively.^41,42^ For the 10 nm Bi on graphene RT depositions, the E_g_ mode is observed at 72 cm^–1^ and the A_1g_ mode at 94 cm^–1^, respectively, also consistent with the β-Bi structure. While the E_g_ band undergoes a blue shift from bulk Bi powder to the 10 nm RT Bi sample, the A_1g_ band for RT depositions shows a slight red shift. This is in accordance with previous studies,^41,43^ suggesting the thin layered nature of Bi deposits obtained during the 10 nm Bi on graphene RT deposition. In the 150 and 250 °C samples, Raman peak intensities reduce below observable significant peaks. This points to low Raman signal due to the observed low coverage (Figure 1b,c and Figure S3) and/or the amorphous nature of the Bi deposits. The amorphous nature of the Bi in the 150 and 250 °C depositions will be confirmed by (S)­TEM below in Figure 5 and 6 and also in Figure S4. Notably, the Raman measurements in Figure 1d exclude the formation of crystalline Bi-oxides from sample preparation or ambient air storage (in accordance with the (S)­TEM analysis below), as bands corresponding to Bi-oxides at predominantly 128, 315, and 461 cm^–1^ remain absent.^44^

In addition to the Bi features, the graphene-related G band at 1587 cm^–1^ and 2D band at 2674 cm^–1^ indicate the preserved high quality of the CVD graphene^33^ substrate post Bi deposition for all temperatures. Notably, no significant graphene defect-related D band intensity was observed at ∼1350 cm^–1^ in any sample,^33^ further implying the presence of a vdW interaction at the Bi/graphene interface, i.e., the absence of covalent Bi/graphene bonding.^4^ Beyond Raman spectroscopy, the high quality of the graphene after Bi depositions is also evidenced by the sharp SAED graphene reflections patterns for all deposition conditions presented below in Figures 3, 4, and S4.^31,32^ The observation of a vdW interface between Bi and graphene is consistent with previously reported density functional theory (DFT)^45^ calculations and photoemission spectroscopic measurements.^20^

**Figure 3:**
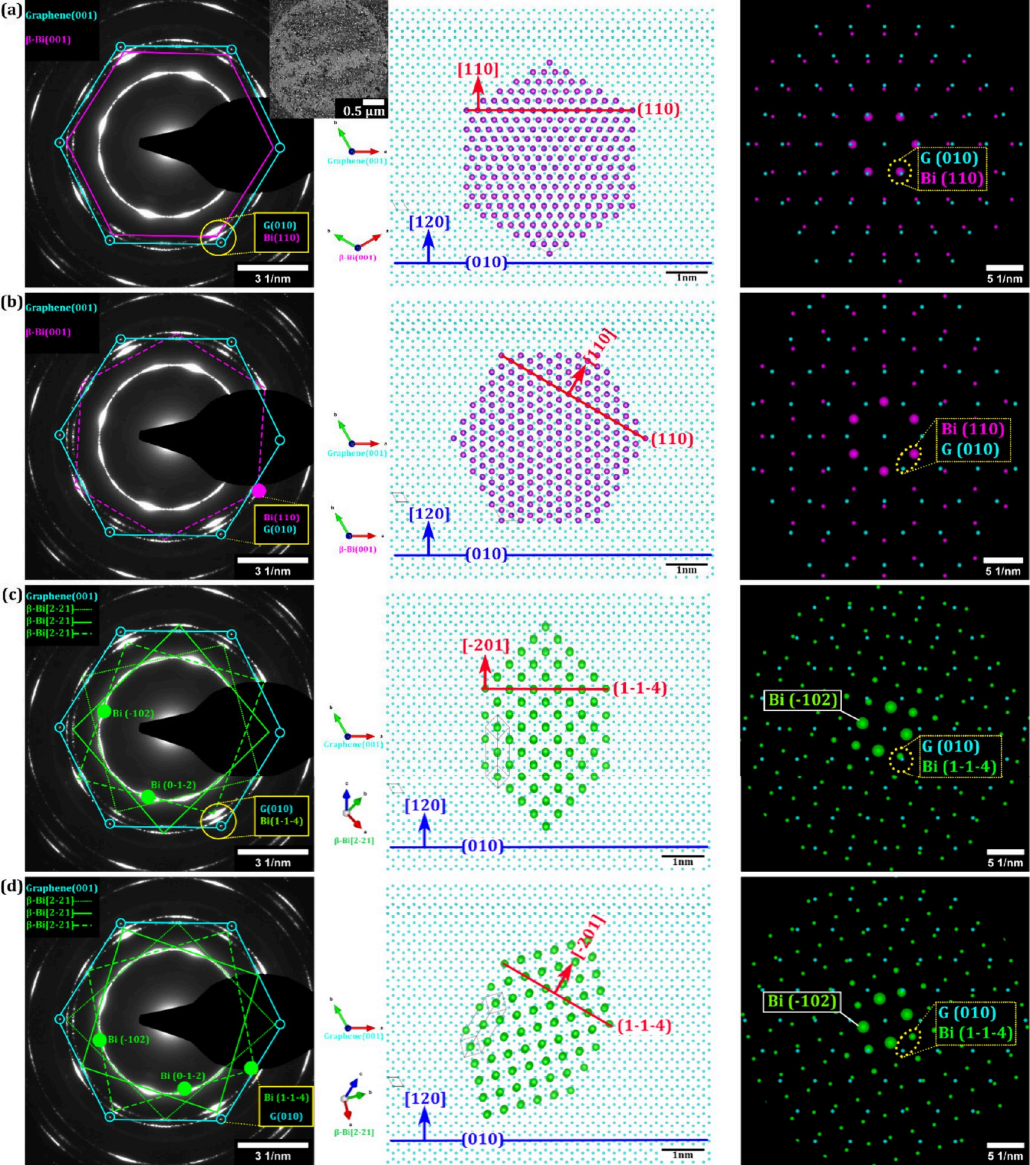
RT Bi depositions (epitaxy). (a, b) β-Bi(001) and graphene indexed SAED patterns (left), atomic models showing in-plane vdW epitaxial relations derived from the corresponding marked features in the SAED patterns (middle), and overlay of simulated ED patterns corresponding to the atomic models and SAED (right). Note that between the atomic models in the middle column and the SAED/ED data in the left/right columns, a rotational offset of ∼30° exists, which is however clarified by the index lattice planes in the middle column and the indexed diffraction spots in the left/right columns. The data suggest the presence of two concurrent epitaxial relations of β-Bi(001) with respect to graphene: β-Bi(001)||graphene(001)/β-Bi[110]||graphene[120] and β-Bi(001)||graphene(001)/β-Bi[110] ∠30° graphene[120]. The inset in (a) presents a BF-TEM image of the region on which the SAED data have been acquired. (c, d) β-Bi[2–21] and graphene indexed SAED patterns (left), atomic models showing in-plane vdW epitaxial relations derived from the corresponding marked features in the SAED patterns (middle), and overlay of simulated ED patterns corresponding to the atomic models and SAED (right). These suggest the presence of two concurrent epitaxial relations of β-Bi[2–21] with respect to graphene as well: β-Bi[2–21]⊥graphene(001)/β-Bi[−201]||graphene[120] and β-Bi[2–21]⊥graphene(001)/β-Bi[−201] ∠30° graphene[120]. The color coding is as follows: magenta = β-Bi(001), green = β-Bi[2–21], cyan = graphene(001). All four SAED patterns in (a–d) are the same and were recorded on the region shown in the (a) inset. The magenta colored ED is indexed to β-Bi viewed along the [001] zone axis. The green colored ED is indexed to β-Bi viewed along the [2–21] zone axis, and the cyan colored ED is indexed to graphene viewed along the [001] zone axis. The unit cells and zone axes for the corresponding elements in the epitaxy atomic models have been indicated. We also note that while referring to impressed epitaxial relations expressed for Bi on graphene, the focus should be on the plane and the direction mentioned in the epitaxial relations and not on the morphology of the β-Bi(001) and β-Bi[2–21] (particularly in the atomic models). The morphology used is for the sake of representation of atomic models and might differ from the exact atomic edge structures. The Bi discrete intensity maxima at discrete rotation angles with respect to the 6-fold graphene spots are best visible at the respective yellow marked reflection sets in (a–d) at ∼0.22 nm due to the wider angular spread for a larger reciprocal distance from the central beam. Additionally, for the reflections at ∼0.3 nm, a halo from the amorphous region 3 from Figure 2 may contribute to the signal background.

**Figure 4:**
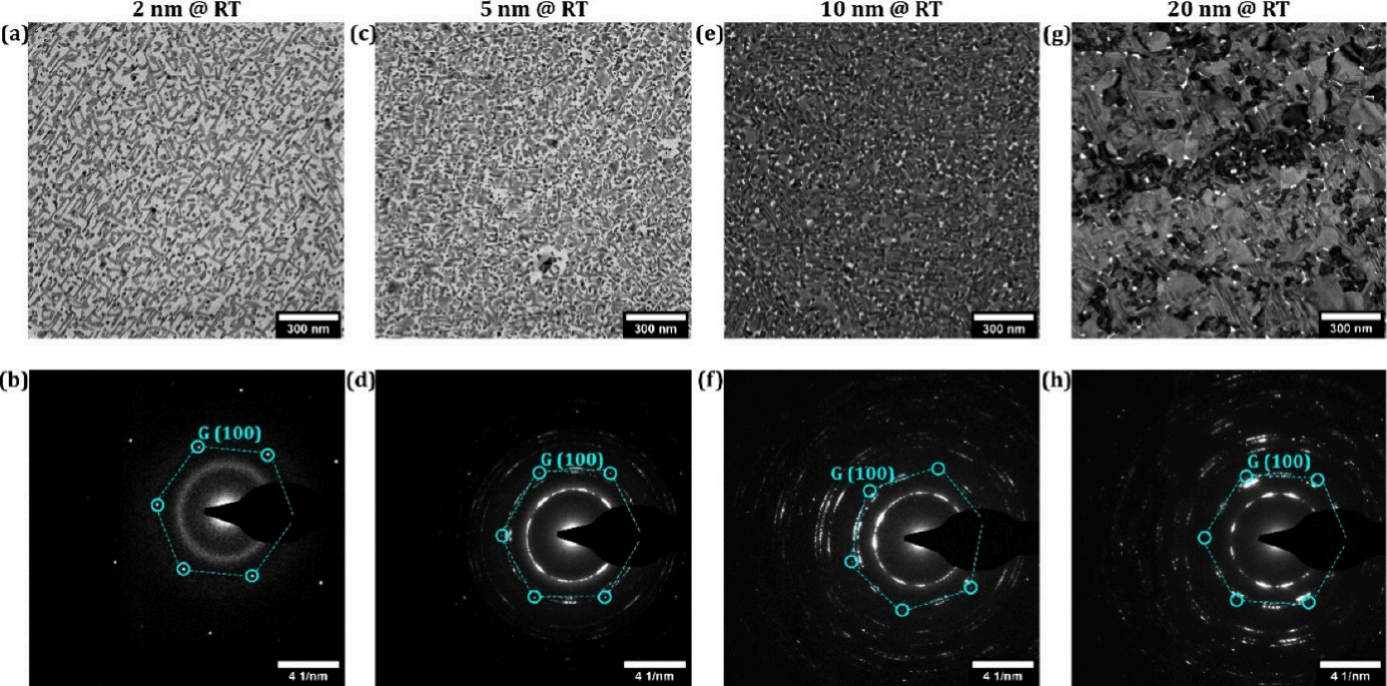
Bi RT growth parameter space. BF-TEM images and corresponding SAED patterns of Bi deposits of thicknesses 2 nm (a, b), 5 nm (c, d), 10 nm (e, f), and 20 nm (g, h) on suspended graphene. The preserved reflections from graphene underneath in the SAED patterns have been marked in cyan color and have been indexed to graphene(001) viewed along the [001] zone axis.

### RT Bi Depositions

For the as deposited 10 nm Bi depositions at RT, films with three regions with different atomic arrangements were observed on the graphene supports, as observed via STEM in Figure 2a,b. First are regions (example marked “1” in Figure 2a–c) showing well-crystallized 6-fold symmetry of Bi atoms in projection (high-resolution STEM analysis in Figure 2c,d–f). This is assigned to the most thermodynamically stable β-Bi allotrope (rhombohedral, *R*3̅*m*, A7) with an out-of-plane texture of the β-Bi[001] zone axis perpendicular to graphene(001) (β-Bi[001]⊥graphene(001)), equivalent to the β-Bi(001) plane parallel to graphene(001) (β-Bi(001)||graphene(001)). Second are regions (example marked “2” in Figure 2a–c) with well-crystallized 4-fold symmetry of Bi atoms in projection (high-resolution STEM analysis in Figure 2c,g–i). This can be assigned to β-Bi with zone axis β-Bi[2–21]⊥graphene(001) texture, which shows a pseudo-cubic symmetry in projection. A very close terminating (but not perfectly parallel) plane for this texture is β-Bi(1–12), see Figure S2. Since β-Bi(1–12) is equivalent to β-Bi(012), this orientation of β-Bi has in earlier literature often been referred to as β-Bi(012).^46^ Notably, in projection this structure is similar to black-phosphorus-like A17 allotrope or α-Bi(001).^46^ We will refer to the two observed β-Bi textures as β-Bi(001) and β-Bi[2–21], respectively, for the remainder of this article. Third are a few regions (example marked “3” in Figure 2a,b) that show a predominantly amorphous structure, which via a close examination by Fourier transform (FT, Figure S5) reveal a short-range order consistent with the prime ∼0.33 nm lattice distance found in β-Bi[2–21]. We therefore assign the amorphous regions as less ordered predecessors (“amorphous β-Bi[2–21]-like”) to the much more crystallized β-Bi[2–21] regions. Beyond the β-Bi(001), β-Bi[2–21], and the few amorphous β-Bi[2–21]-like regions, no signs of other phases, e.g., Bi-oxides, have been detected by (S)­TEM,^39^ which is in good agreement with the Raman data above. We also note that the RT depositions did not show any e-beam induced^31,32^ structure or morphology changes under our imaging conditions. See Figure S2 for simulated ED patterns of β-Bi(001), β-Bi[2–21], and graphene(001) for reference against the experimental atomic-resolution data in Figure 2.

In terms of morphology, in Figure 2a,b the 6-fold symmetric β-Bi(001) and the amorphous β-Bi[2–21]-like regions are predominantly of irregular shape, while the 4-fold symmetric β-Bi[2–21] are predominantly of one-dimensional rod-like shapes. Figure 2b,c, however, shows that these β-Bi[2–21] rods do not terminate atomically perfectly and have several step edges in their terminal line projections. We also note that some rod-like appearing regions are also of the 6-fold symmetric β-Bi(001) structure.

What is readily apparent in Figures 1a and 2a is that the rod-like deposits appear to have a preferred orientation of their long axes with respect to each other across the entire field of view. Given that the graphene grains are micrometer-sized,^4^ and thus the fields of view in Figures 1a and 2a are likely single single-crystalline graphene grains, this apparent preferred orientation of Bi deposits could be indicative of rotational vdW epitaxy between the Bi and the graphene.^4,31,32^

In Figure 3 we further explore this notion of possible vdW epitaxy. In particular, the Figure 3a inset shows a BF-TEM image of a 10 nm RT Bi film on graphene with its corresponding SAED pattern plotted in the left panels of Figure 3a–d (the same SAED pattern is replotted four times to clearly illustrate the four different epitaxy assignments). The highly sharp, 6-fold SAED pattern for the graphene in Figure 3a–d (cyan indexing, showing no change in sharpness compared to the state before Bi deposition)^31,32^ indicates the excellent preservation of the graphene structure upon Bi PVD.^31,32^ This corroborates the formation of a vdW interface between graphene and Bi.^31,32^ Notably, the graphene-related SAED spots in Figure 3a–d (cyan indexing) are composed of only one single 6-fold spot set. This confirms that in the field of view in the Figure 3a inset, only one individual single-crystalline graphene grain has been imaged. This allows us to readily disentangle possible vdW epitaxial effects between the Bi deposits and the supporting single-crystalline graphene grain via SAED and BF-TEM.

Indexing of the SAED in Figure 3a–d with respect to Bi indicates a mixture of β-Bi(001) (Figure 3a,b, magenta indexing) and β-Bi[2–21] (Figure 3c,d, green indexing) crystalline deposits, in accordance with the STEM data in Figure 1 (see also Figure S2 for reference of simulated ED patterns). Importantly, Figure 3a–d shows that the reflections corresponding to β-Bi(001) (magenta indexing) and β-Bi[2–21] (green indexing) are not randomly arranged (forming rings) but instead have discrete intensity maxima at discrete rotation angles with respect to the 6-fold graphene spots. This periodic angular intensity distribution is a telltale sign of rotational vdW epitaxy between the graphene and the Bi deposits.^4,32^

In particular, we measure in Figure 3a for β-Bi(001) a first preferred orientation of the β-Bi(001) [110] direction with no rotational offset to the graphene [120] direction (β-Bi(001)||graphene(001) and β-Bi[110]||graphene[120]). In Figure 3b, we measure a second preferred orientation of the β-Bi(001) [110] direction with a 30° offset to graphene[120] (β-Bi(001)||graphene(001) and β-Bi[110] ∠30° graphene[120]). In Figure 3c,d we measure for β-Bi[2–21] several equivalent sets of spots. We note that due to the respective 6-fold symmetry of graphene, this however translates to just two relative preferred β-Bi[2–21] crystal lattice orientations (including mirroring) with respect to the graphene lattice. The first is in Figure 3c: the [−201] of β-Bi[2–21] with no rotational offset to graphene[120] (β-Bi[2–21]⊥graphene(001) and β-Bi[−201]||graphene[120]). The second is in Figure 3d: the [−201] of β-Bi[2–21] with a 30° offset to graphene[120] (β-Bi[2–21]⊥graphene(001) and β-Bi[−201] ∠30° graphene[120]). Thus, our data clearly establish that rotational vdW epitaxy exists in the Bi/graphene system for both β-Bi(001) and β-Bi[2–21], even in the case of nonsupported, free-standing graphene. The rotational vdW epitaxial relationships arise from weak van der Waals interactions between Bi and graphene, allowing remote registry without covalent bonding.^3^ Thermodynamically, these alignments minimize interfacial energy by achieving quasi-commensurate lattice matching.

Figure 4 expands our investigation to studying the evolution of Bi RT depositions as a function of deposited Bi amount by investigating varying nominal thicknesses from 2 to 20 nm. The highly sharp, 6-fold SAED patterns for the graphene grains in Figure 4b,d,f,h again indicate the excellent preservation of the graphene structure upon Bi PVD, again corroborating a vdW Bi/graphene interface. Notably, the graphene-related SAED spots in Figure 4b,d,f,h are all predominantly composed of only a single 6-fold spot set, indicating that in each BF-TEM image in Figure 4a,c,e,g, only single single-crystalline graphene grains have been imaged.

For the initial 2 nm nominal thickness, Figure 4a shows that the Bi structures nucleate homogeneously over the entire monolayer graphene basal plane without yet forming a closed film (areal coverage of ∼54%, see Figure S3b). Interestingly, the SAED in Figure 4b indicates that at this stage, the Bi deposit is of a mostly amorphous nature. Closer inspection of the ring-like halo in Figure 4b at ∼0.33 nm reveals that this is again consistent with the amorphous β-Bi[2–21]-like structure similar to the region example marked “3” observed in the 10 nm RT depositions, as shown in Figures 2a,b and S5. The small, darker contrast particles in Figure 4a are identified as small crystals with β-Bi[2–21] structure (Figure S3c). The morphology indicates at this stage already coexisting rod-like shapes and irregular shapes of deposits. Notably, the rod-like deposits appear to have preferred orientation alignment over the field of view in Figure 4a. Interestingly, this is despite the mostly amorphous nature of the deposits.

When increasing the Bi deposit thickness to 5 nm, in Figure 4c the morphologies of features remain of the same nature, but they cover a higher fraction of the substrate, slowly closing toward a closed film (areal coverage of ∼73%, see Figure S3b). Again, preferred orientations of the rod-like deposits are evident in the field of view in Figure 4c. Notably, the SAED in Figure 4d shows that at this stage, the Bi film has crystallized. Indexing of the SAED in Figure 4d indicates a phase mixture of β-Bi(001) and β-Bi[2–21] with the same vdW epitaxy relations as those discussed above.

Upon reaching a 10 nm nominal thickness in Figure 4e, an almost fully covering film (akin to Figures 1a, 2a, and 3a, inset) is formed (areal coverage of ∼95%, see Figure S3b). Consistent with the 5 nm deposition, the SAED in Figure 4f indicates the presence of both β-Bi(001) and β-Bi[2–21] on the graphene and also exhibits the same rotational vdW epitaxial orientations as those described above.

Upon reaching a 20 nm nominal thickness in Figure 4g, the almost fully covering film (areal coverage of ∼99%, see Figure S3b) has visually grown in overall thickness, evidenced by the lower intensity in the BF-TEM image. Notably, we observe for the 20 nm film a somewhat larger fraction of irregularly shaped Bi grains compared to a reduced number of (albeit longer) rod-like deposits. Again, β-Bi(001) and β-Bi[2–21] are detected in the SAED in Figure 4h with the same rotational vdW epitaxy relations as above. Based on the above established morphology–structure relationship and the SAED in Figure 4h, the increased fraction of irregularly shaped regions in Figure 4g points to a larger fraction of β-Bi(001) in the 20 nm film compared to the thinner films.

While our work is the first report on Bi deposition on truly freestanding graphene, we now set our observations in context with prior work on Bi deposition on supported graphene^20–22^ and graphene’s bulk analogue graphite.^23,25^ Prior work has observed for RT depositions mixed β-Bi(001)/β-Bi[2–21][Bibr ref21] and exclusive β-Bi[2–21] ^20,22^ growth on supported graphene. Likewise, for bulk graphite, β-Bi(001), β-Bi[2–21], and mixed growth^23,25^ have been reported for RT depositions. Morphologies of the β-Bi(001) and β-Bi[2–21] varied depending on study, but β-Bi(001) has been reported to exhibit (triangular) island^23^ morphology, somewhat matching our observed irregular shapes, while β-Bi[2–21] was reported as rod-like,^24^ consistent with our observations, and star-shaped.^23,24^ For the planar freestanding monolayer graphene, we observe homogeneous nucleation on the graphene basal plane. Due to the absence of step edges on our freestanding monolayer graphene films, clearly no preferential nucleation at steps could be observed, as is often seen in work on bulk graphite with ample step edges.^24^

Prior work has not observed an amorphous β-Bi[2–21]-like deposit as a third morphology, as we do exclusively in the 2 nm depositions and in small fractions also in the consecutive higher nominal thickness depositions such as the 5 and 10 nm, as shown in Figures 2b and S5. The initial growth of Bi in amorphous β-Bi[2–21]-like form at ultralow thicknesses, which then undergoes crystallization upon increasing Bi thickness, has not been reported for Bi before,^23^ but such a mechanism has been established for closely related Sb.^47^ We suggest that this observation here may be related to the truly freestanding graphene support, which is in contrast to all prior work on supported or bulk supports. A transition from β-Bi[2–21] to β-Bi(001), i.e., a higher β-Bi(001) fraction with increasing Bi deposit thickness, has been previously reported, consistent with our 20 nm data. This subsequent transition of β-Bi[2–21] to β-Bi(001) is due to the low thickness stability of β-Bi[2–21].^23,46^

Prior studies investigating the growth of Bi on similar adopted substrates like supported graphene on SiC^20^ and HOPG^23,24^ have observed identical epitaxial relations of the grown Bi structures with respect to the graphene underneath, as we observe and discuss above. This suggests that further SiC and HOPG “bulk” supports underneath an individual graphene layer (as here) do not change the intrinsic vdW epitaxy relationships for Bi deposits.

### Higher Substrate Temperature Bi NPs Including E-Beam Driven Dynamics

In contrast to the crystalline, film-like nature of the RT depositions with an areal coverage of ∼95% (Figure S3) for nominal 10 nm depositions, the nominal 10 nm depositions at 150 and 250 °C substrate temperatures resulted in isolated Bi NPs (Figure 1b,c) with sizes ranging from 3 to 20 nm (see Figure S4d) and a much reduced areal coverage of ∼3% and 0.4% at 150 and 250 °C, respectively (see Figure S3). TEM shows that these NPs are largely amorphous in the deposited state (Figure 5a,b, 00 min and Figure S7, 00 min), showing a subtle substructure of discrete amorphous, adjacent islets comprising the individual particles. A thickness estimation based on HAADF STEM intensity analysis^31,32^ places the average thickness of the 250 °C particles at ∼2 nm (see Figure S3a). This translates to a loss of Bi amount between the RT and 250 °C depositions of ∼99%.

**Figure 5:**
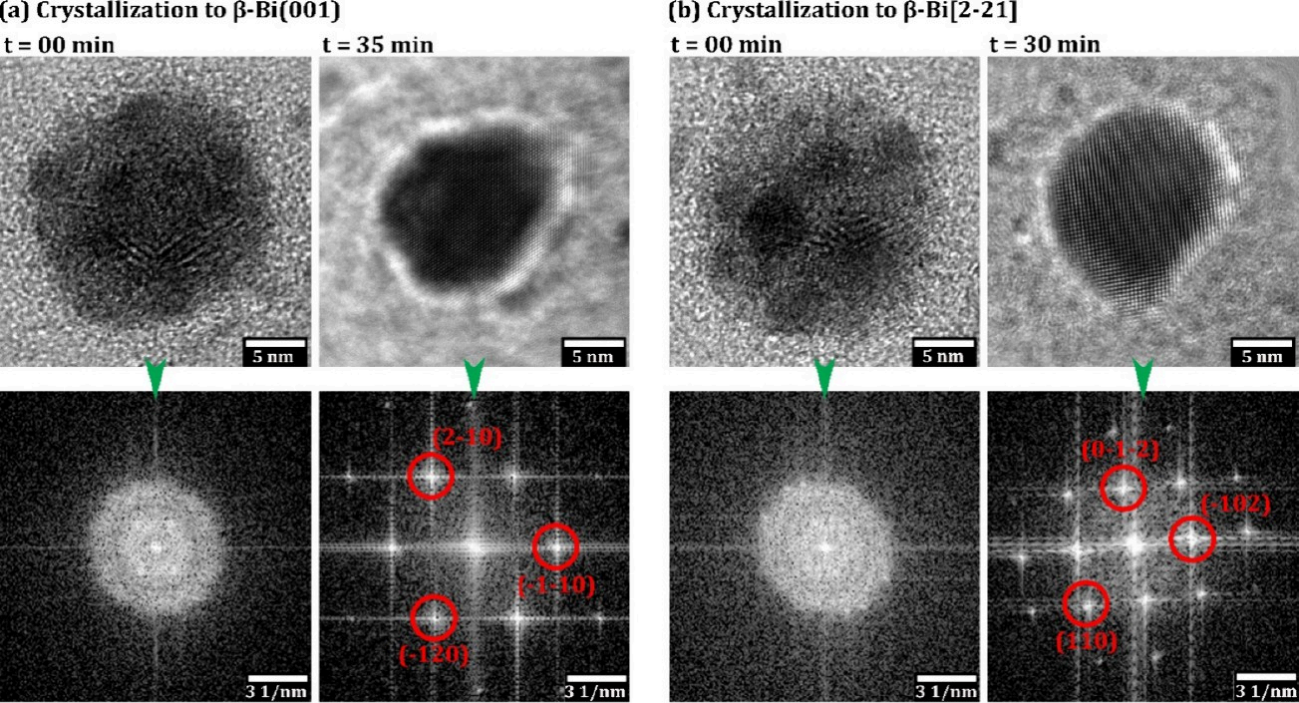
Bi 150 °C *in situ* crystallization. BF-TEM images of electron beam induced crystallization of amorphous Bi NPs obtained from 150 °C deposition. (a) The crystallization of thicker (based on BF-TEM contrast) amorphous deposits to β-Bi(001) and (b) the crystallization of thinner (based on BF-TEM contrast) amorphous deposits to β-Bi[2–21]. The FT patterns of the BF-TEM images of the crystallized NPs have been indexed as per simulated ED patterns as shown in Figure S2.

With a melting point of bulk Bi at ∼270 °C,^48^ the drastic morphology change as observed in Figure 1a–c in the Bi from RT to higher temperature depositions is related to nanosize melting point depression at the higher temperature depositions (to, for example, ∼160 °C for 5 nm Bi particles)^49^ and the associated much increased backward Bi desorption during deposition at higher substrate temperatures.^4,38^ While our RT films are crystalline, the 150 and 250 °C deposits are found to be amorphous. The amorphous structure results from cooling from the above-melting-point substrate temperature of 150 to 250 °C, whereby the natural cooling in a vacuum as used here is found to be sufficient to result in the amorphous structure, suggesting that no active fast quenching beyond natural cooling is necessary to obtain this amorphous structure. Prior work has observed for the annealing of RT deposited Bi films or high temperature Bi deposition a similar reduction of retained Bi,^25,50,51^ with very few examples of the retention of crystallinity at 150 °C albeit with restructuring of morphology or oxidation.^25^

In contrast to the static nature of the RT Bi depositions under the e-beam in (S)­TEM and TEM, as observed above, the Bi NPs from the higher temperature 150 and 250 °C depositions show a great deal of atomic motions upon e-beam exposure in TEM under our conditions, as shown in Figure 5a,b (electron dose rate estimated to 1 × 10^2^ e^–^ Å^–2^ s^–1^). In particular, the amorphous nature of the Bi NPs from the higher temperature depositions changed *in situ* into fully crystallized structures with a faceted morphology. We find that the amorphous NPs can readily evolve into NPs with either β-Bi(001) (Figure 5a) or β-Bi[2–21] (Figure 5b) structure.

We find in our *in situ* measurements that the crystallization of the initially amorphous Bi NPs proceeds via a two-step mechanism: In step 1, the crystallization begins with the formation of several irregularly shaped, isolated crystallized clusters (shown with magenta (for β-Bi(001) and green (for β-Bi[2–21]) markup regions) within the amorphous islets of Bi NPs (Figure 6a, 0–25 min; Figure 6b, 0–18 min). In step 2, these initially crystallized regions within the particles coalesce with each other and continue to rearrange, ultimately leading to the gradual formation of a faceted crystal (Figure 6a,b). The final crystalline Bi islet formed has a reduced projected area (and thereby a higher density and/or thickness) as compared to the initial amorphous particles (see Figure S6). Once crystallized, the Bi particle remains crystallized during further longer electron beam exposure, until electron beam induced damage^31^ to the supporting graphene limits the stability of the entire heterostructure. We find that some amorphous particles could take as short^52^ as ∼3–4 min to completely crystallize under our conditions.

**Figure 6:**
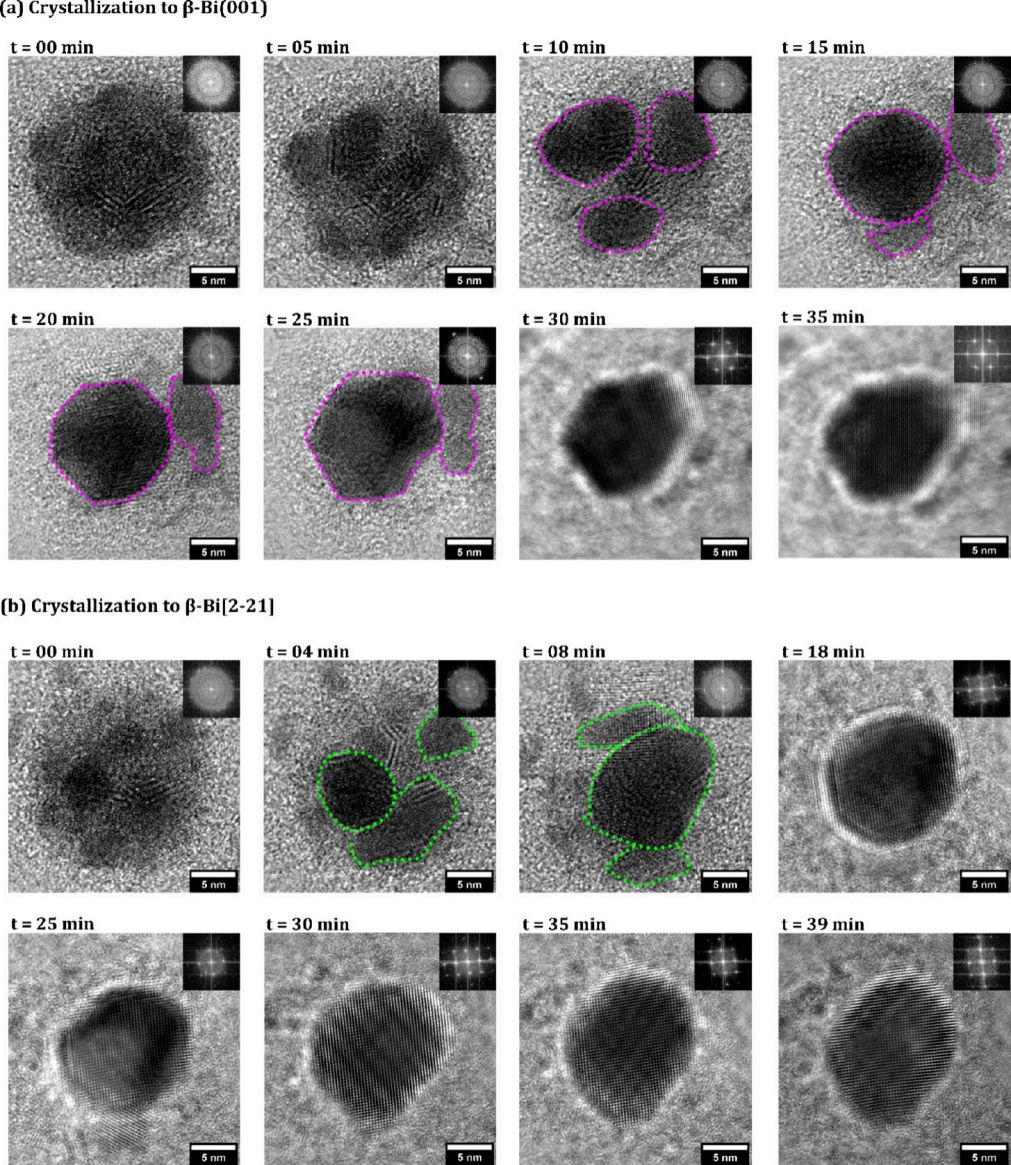
Bi 150 °C *in situ* crystallization time series. BF-TEM images showing various microscopic stages during electron beam induced crystallization of amorphous Bi NPs obtained from 150 °C deposition at different time stamps. (a) The crystallization pathway of thicker (based on BF-TEM contrast) amorphous deposits to β-Bi(001) as in Figure 5a. (b) The crystallization pathway of thinner (based on BF-TEM contrast) amorphous deposits to β-Bi[2–21] as in Figure 5b. The FT patterns on the BF-TEM images of the crystallized NPs at different time scales are shown in the insets.

The crystallization mechanism here seems to follow the nonclassical pathway of cluster coalescence mediated crystallization by particles. This is in line with the observation made on amorphous Bi nanosheets, where the phase transformation of Bi metal from amorphous to crystalline structure takes place via the particle mediated nonclassical mechanism instead of the classical atom mediated mechanism.^53^ We note that the observed crystallization pathways of our amorphous Bi NPs can be classified as “nonclassical”^54^ because they are characterized by cluster coalescence rather than by classical atom-by-atom nucleation. In classical nucleation theory,^54^ crystal growth proceeds via monomer attachment to a critical nucleus, driven by thermodynamic minimization of the Gibbs free energy through bulk phase stability. However, in nanoscale systems like our Bi NPs, nonclassical mechanisms can dominate due to kinetic factors, such as high surface-to-volume ratios and low diffusion barriers for preformed amorphous clusters.^54^ The driving force hereby may primarily be the reduction in surface energy: amorphous islets within the Bi NPs exhibit disordered, high-energy interfaces that favor rapid coalescence into ordered clusters, lowering the overall interfacial energy and facilitating the stepwise rearrangement into faceted Bi NP crystals, as observed here. In our experiments, this process is kinetically enabled by the energy input from the electron beam, which acts as a proxy for thermal annealing, overcoming activation barriers without actually reaching bulk melting temperatures.^31,32^

Within our data, apparently thicker amorphous Bi islets (based on the intensity in the BF-TEM images) show a preferential transition to β-Bi(001) based on the high thickness stability of β-Bi(001), as outlined by several prior seminal works.^46^ In contrast, thinner amorphous Bi islets preferentially crystallize to β-Bi[2–21]. Overall, we observe more crystallization events to β-Bi[2–21] (∼40) than to β-Bi(001) (∼9) for the on average ∼6 nm thick Bi NPs obtained in 150 °C deposition. We also observe events of two isolated islets coalescing into each other and ultimately crystallizing to β-Bi(001) (Figure S7). These observations are in line with the transition of preference from β-Bi[2–21] to β-Bi(001) at higher thicknesses,^23,46^ akin to the RT deposition samples. Figure S8 captures even finer details of such beam induced crystallization events in Bi NPs at a higher temporal resolution. The same can also be visualized in Video V1. With respect to rotational vdW epitaxy, we note that for our data set of *in situ* crystallized NPs, we can compare the relative orientation of Bi and graphene lattices via FT analysis post crystallization, as shown for one β-Bi(001) particle in Figure S9a,b and for one β-Bi[2–21] particle in Figure S9d,e. From the multiple (>45) crystallization sequences, we can plot a histogram of rotation values between Bi and graphene crystals (Figure S9c for β-Bi(001) and Figure S9f for β-Bi[2–21]). Consistent with the SAED analysis in Figure 3 (which intrinsically averaged over multiple >140 crystallites), this statistical analysis of single particles also shows signs of rotational vdW epitaxy for Bi/graphene. For β-Bi(001), we find in Figure S9c a preferred orientation of the β-Bi(001) [110] direction with no rotational offset to the graphene [120] direction (β-Bi(001)||graphene(001) and β-Bi[110]||graphene[120]), akin to Figure 3a. For β-Bi[2–21], we find in Figure S9f the [−201] of β-Bi[2–21] with no rotational offset to graphene[120] (β-Bi[2–21]⊥graphene(001) and β-Bi[−201]||graphene[120]), akin to Figure 3c. The absence of 30° misorientations with respect to graphene[120] (as in Figure 3b,d) for both β-Bi(001) and β-Bi[2–21] may either be related to the smaller number of observations than in the multicrystallite (>140) averaging SAED in Figure 3 or indicates that the emergence of certain rotational vdW epitaxy relations in the Bi/graphene can also be kinetically limited during deposition vs recrystallization pathways.^31,32^

Electron beam exposure leading to amorphous/crystalline transitions in Bi NPs has been previously reported unidirectionally^53^ (as here) and also in a reversible manner.^55^ It has been suggested to be related to heating/supercooling of the Bi particles via the energy delivered by the electron beam due to different thermal conductivities in the crystalline/amorphous states of Bi,^52^ which can lead to cyclic amorphization/crystallization of Bi NPs. (The exact mechanism of energy delivery from the e-beam to the Bi is beyond the scope of this study but may also include knock-on or radiolysis-type effects,^56^ rather than an actual temperature rise.)^31^ In our data, we did only observe forward amorphous to crystalline transitions, while cyclic crystallized ↔ amorphous state transitions were not observed. The reverse transformation of the crystalline to amorphous state in Bi has thus far been attributed to continued heat input from the electron beam into the Bi, which results in reaching the nanosize-related depressed melting point and thus causes liquefaction of the Bi crystallites under the beam.^52^ We suggest that the fact that we do not observe this liquefaction behavior for our Bi crystallites could be related to the efficient heat transfer away from the Bi because of the excellent heat conducting properties of the graphene membrane. Thus, due to the graphene support, we do not reach the threshold for the reverse liquefaction transition (before the graphene support loses structural integrity from beam damage for very prolonged e-beam exposures).

The observation of ∼45% area reduction upon complete crystallization of the amorphous Bi NPs further overlaps with the initial liquid (amorphous) state to final solid (crystallized) state, where compact structuring of the Bi atoms in their crystallized state leads to the reduction in area of the Bi NPs (see Figure S6).

### Comparison of Bi and Sb on Graphene Heterostructures

We now set our here presented results on Bi/graphene heterostructures in context with our recently published parallel (S)­TEM-based investigation of few-layer antimonene (2D Sb)/graphene heterostructures.^4^ Notably, Sb (atomic number 51) is the pnictogen directly above Bi (atomic number 83) in group 15/VA in the periodic table. Key differences between Sb and Bi are the much higher melting temperature of Sb of ∼630 °C compared to that of Bi of ∼270 °C,^57^ as well as the more anisotropic, layered character of Sb compared to that of Bi.^58,59^ A common observation in both Sb and Bi on graphene systems is the general coexistence of the two (001) and [2–21] textures in crystalline deposits.^4^ Also, both Sb/graphene and Bi/graphene systems show clear rotational vdW epitaxy.^4^ This shows that both pnictogens form structurally similar heterostructures with graphene. The higher melting temperature of Sb, however, translates to the amorphous Sb growth at RT^4^ vs the fully crystallized Bi growth at RT for the same nominal thickness. Likewise, the retained amount of Sb at higher deposition temperatures up to 250 °C is much higher^4^ than the here observed drastic Bi loss. Also, the higher temperature Sb depositions readily retain crystallinity,^4^ unlike the here amorphized Bi NPs at 150 and 250 °C. Combined, this clearly shows that processing windows to obtain a similar pnictogen structure on graphene must differ in temperature and flux. A final difference between Sb and Bi was that, unlike in the Bi system where amorphous NPs showed electron beam induced crystallization phenomena, in the Sb system all nanostructures remained static under the electron beam in our employed (S)­TEM conditions.^4^ We again ascribe this to the higher melting point of Sb compared to that of Bi. This information has also been tabulated in a summarized form in Table S1.

### Plasmon Shift during *In Situ* Crystallization

Finally, in addition to following the structural evolution of the initially amorphous 150 and 250 °C deposited Bi NPs *in situ* under the electron beam in Figures 5 and 6, we also follow the evolution of Bi NP plasmon signatures using time-resolved (V)­EELS. Recent advances in the instrumentation along with adapted mathematical methods have made the use of time-resolved EELS in studying electron beam induced phase transformations quite feasible. However, most time-resolved EELS studies remain concentrated on the core-loss spectra. Hardly any of the time-resolved studies have focused on the change in the low loss region (with special emphasis on the shift of the plasmonic modes) to account for the electron beam induced phase transitions.^60^ Given the key interest in plasmonic properties of Bi NPs^10^ for optical switching and plasmon catalysis applications and given the possible key influence of structure modifications on plasmonic signatures, such measurements would, however, be of high relevance. The measurements were performed in TEM mode with the electron dose rate estimated to 3 × 10^3^ e^–^ Å^–2^ s^–1^, which led to the complete crystallization of Bi NPs in 4 min, unlike the electron dose rate of 1 × 10^2^ e^–^ Å^–2^ s^–1^ above, which took ∼30 min to crystallize the NPs.

In Figure 7 we show a starting VEELS spectrum of an amorphous Bi particle on graphene (i.e., a Bi particle from 150 °C higher substrate temperature deposition) but for which the EELS contribution of graphene has been subtracted as background, i.e., Figure 7a shows only the Bi-related VEELS signal. A fit of the VEELS spectrum reveals three components, namely a predominant peak centered at ∼19 eV, which is ascribed to the Bi volume plasmon (VP),^61^ a shoulder to this peak at ∼10 eV, which is ascribed to a surface plasmon (SP) of the Bi NP,^61^ and another peak at ∼29.9 eV, which is related to the Bi O_4,5_ edge.^61^ These VEELS features are as expected for Bi nanostructures,^61^ also further excluding the formation of other phases such as Bi-oxides, in line with the Raman and (S)­TEM data above.^62^

**Figure 7:**
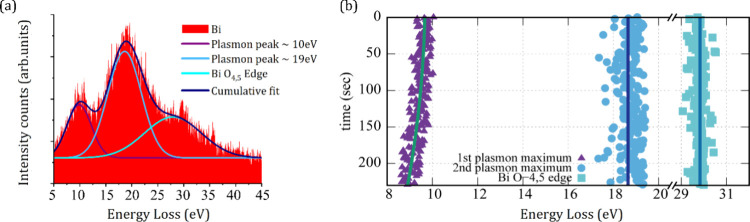
Plasmon shift during *in situ* crystallization. *In situ* studies of Bi plasmon evolution during electron beam induced crystallization of Bi NPs on graphene at 150 °C. (a) Low loss EELS spectrum of Bi composed of three distinct features. The peak at ∼10 eV corresponds to Bi SP. The peak at ∼19 eV corresponds to Bi VP, and the peak at ∼30 eV is the Bi O_4,5_ edge. (b) Time-resolved evolution of the three distinct features of the Bi low loss EELS spectrum during electron beam driven crystallization. The Bi SP peak undergoes a shift from the initial ∼10 eV (amorphous state) to a final ∼8.6 eV (crystallized state), while there are no significant changes in the ∼19 eV VP peak position and the Bi O_4,5_ edge.

Previous studies on Bi NPs and thin films have reported the VP energy in a range of ∼14 ^61,63^ to ∼19 eV.^64^ An increase in the VP energy has been reported with decreasing particle size, which is attributed to quantum confinement effects in Bi NPs. Specifically, as the size of Bi NPs decreases below 40 nm, the experimentally observed VP energy increases. Similar trends have also been observed in other Bi nanostructures such as thin films, nanowires, and nanorods.^65^ Wang et al. reported a VP energy of 19.8 eV for Bi NPs with a lateral size of 11 nm.^64^ In our experiments, Bi NPs synthesized at 150 °C have lateral sizes of ∼11–13 nm. Consistent with previous observations, the VP energy of our Bi NPs is measured to be at ∼19 eV. Electromagnetic simulations in Figure S10 further reaffirm that the peak observed in Figure 7a at ∼10 eV is an SP.

In Figure 7b we plot how the energies of the three components (VP, SP, and Bi O_4,5_) change with increasing exposure time to the e-beam during the Bi NP transition from the initial amorphous to final crystalline Bi in a time period of 4 min. Notably, while the VP peak at ∼19 eV and Bi O_4,5_ edge at ∼29.9 eV do not change in energy throughout the crystallization process, the SP shoulder at ∼10 eV downshifts with increasing e-beam exposure time to a lower energy of ∼8.6 eV. The shift in SP suggests that the Bi SP is sensitive to the crystallization process of the Bi NPs.

The dependence of VP and SP energies on the metal NP (i.e., not only Bi-specific) crystallization state^60^ can often be coupled with accompanying changes in NP size^66^ and can be complex (e.g., even trend reversals for SP energies as a function of NP size).^66^ For instance, prior work showed that crystallization from amorphous to crystalline led to changes in the VP position for Sn NPs^60^ and in the SP signature for GeTe NPs.^67^ Here, we find for our Bi NPs the VP position to be insensitive to crystallization and the SP to downshift to lower energy upon crystallization.

Correlating with the *in situ* evolution in TEM in Figures 5 and 6, we see that our Bi NPs during crystallization become more compact, leading to a decrease in lateral NP size (see above and also Figure S6). Based on prior reports on the general NP size dependence of SPs in metal NPs^66^ (i.e., not Bi-specific) for such a lateral NP size decrease, an upshift of SP energy to higher energy would be expected. We experimentally, however, observe in Figure 7b a downshift to lower energy (i.e., the opposite direction as expected for the observed NP size reduction from the metal NP model).^66^ This suggests that the observed SP energy downshift in Figure 7b is related to the observed Bi NP crystallization, which is the second key parameter changing from the *in situ* e-beam exposure besides particle size.

We have probed the SP and VP evolution *in situ* for Bi on freestanding graphene, which no prior work has done before. Also, no prior work has measured the SP and VP evolutions *in situ* for one given Bi nanoparticle during crystallization (as we do here for the first time). To date, prior works have only measured VP size dependence from pre-existing nanoparticles of different sizes but without changes to their crystalline state.^61,63–65^ Comparison to prior SP trends based on particle size models for metal NPs in general (not Bi-specific)^66^ suggests that the here observed trend in SP energy is opposite to what is expected for size evolution and thus is suggested to be related to the second key change (besides size), which is the crystalline state of the Bi. We hope that this suggestion stimulates further studies (e.g., density functional theory studies) into this since Bi is also currently emerging as a plasmonic material of high interest.^10^

Prior studies have shown that Bi possesses a solid–liquid phase transition that can markedly modify its dielectric function.^68^ While some previous works^9,17,69,70^ have described the change in Bi NP optical responses to be a result of a change in dielectric function as well as transmission and reflection changes during the melting and freezing of the Bi in different matrices, to the best of our knowledge no EELS-based study on the effect of the crystallization process on the plasmonic modes of Bi has been reported to date. Since crystallization can be enforced by external stimuli and has been shown in prior work to be cyclic reversible,^55^ this finding may point to the Bi surface plasmon as a potentially switchable plasmonic feature in future works.

## Conclusions

In summary, we studied here the structure and morphology of low-dimensional Bi deposits on truly freestanding monolayer graphene membranes (i.e., removing any possible effect of an underlying substrate under the supported graphene). Our high-resolution (S)­TEM investigations of mixed-dimensionality Bi/graphene heterostructures revealed the structure and morphology evolution of PVD Bi as a function of substrate temperature and deposition amount during deposition, where the blanket-like, largely crystalline thin film morphology under RT conditions drastically transformed to isolated amorphous NPs at higher substrate temperatures. Nucleation of crystalline β-Bi at RT appears to be preceded by an intermediate amorphous state at ultralow thicknesses. The coexistence of two key β-Bi textures was observed in all crystalline depositions, both with rotational vdW epitaxy with the graphene support. For amorphous Bi particles we revealed electron beam induced *in situ* crystallization in TEM and suggest a link between the crystallization state and surface plasmon energy in Bi by concurrent (V)­EELS measurements. Combined, our findings elucidated the multifaceted structural and morphological dynamics in mixed-dimensionality Bi/graphene heterostructures at high resolution and for truly freestanding graphene.

## Supplementary Material





## Notes

Additional microscopic and spectroscopic characterization data, simulation data, data analyses, and comparison of Sb vs Bi PVD on graphene (PDF)
Video V1: crystallization of Bi nanoparticles under electron beam at high temporal resolution (MP4)

## References

[1] Pumera M., Sofer Z. (2017). 2D Monoelemental Arsenene,
Antimonene, and Bismuthene: Beyond Black
Phosphorus. Adv. Mater..

[2] Jariwala D., Marks T. J., Hersam M. C. (2017). Mixed-Dimensional van Der Waals Heterostructures. Nat. Mater..

[3] Periwal P., Thomsen J. D., Reidy K., Varnavides G., Zakharov D. N., Gignac L., Reuter M. C., Booth T. J., Hofmann S., Ross F. M. (2020). Catalytically Mediated
Epitaxy of
3D Semiconductors on van Der Waals Substrates. Appl. Phys. Rev..

[4] Gupta T., Elibol K., Hummel S., Stöger-Pollach M., Mangler C., Habler G., Meyer J. C., Eder D., Bayer B. C. (2021). Resolving Few-Layer
Antimonene/Graphene Heterostructures. npj 2D
Mater. Appl..

[5] Hoffman C. A., Meyer J. R., Bartoli F. J., Di Venere A., Yi X. J., Hou C. L., Wang H. C., Ketterson J. B., Wong G. K. (1993). Semimetal-to-Semiconductor Transition
in Bismuth Thin
Films. Phys. Rev. B.

[6] Freitas R. R. Q., Rivelino R., de Brito
Mota F., de Castilho C. M. C., Kakanakova-Georgieva A., Gueorguiev G. K. (2015). Topological
Insulating Phases in Two-Dimensional Bismuth-Containing Single Layers
Preserved by Hydrogenation. J. Phys. Chem. C.

[7] Cheng L., Liu H., Tan X., Zhang J., Wei J., Lv H., Shi J., Tang X. (2014). Thermoelectric Properties of a Monolayer Bismuth. J. Phys. Chem. C.

[8] Zhou J., Chen J., Chen M., Wang J., Liu X., Wei B., Wang Z., Li J., Gu L., Zhang Q., Wang H., Guo L. (2019). Few-Layer Bismuthene with Anisotropic
Expansion for High-Areal-Capacity Sodium-Ion Batteries. Adv. Mater..

[9] Cuadrado A., Toudert J., Serna R. (2016). Polaritonic-to-Plasmonic Transition
in Optically Resonant Bismuth Nanospheres for High-Contrast Switchable
Ultraviolet Meta-Filters. IEEE Photonics J..

[10] Foltýn M., Šikola T., Horák M. (2025). Bismuth Plasmonic Antennas. ACS
Nano.

[11] Yu X., Liang W., Xing C., Chen K., Chen J., Huang W., Xie N., Qiu M., Yan X., Xie Z., Zhang H. (2020). Emerging 2D
Pnictogens for Catalytic Applications:
Status and Challenges. J. Mater. Chem. A.

[12] Dong F., Xiong T., Sun Y., Zhao Z., Zhou Y., Feng X., Wu Z. (2014). A Semimetal
Bismuth Element as a
Direct Plasmonic Photocatalyst. Chem. Commun..

[13] Guo Y., Pan F., Ye M., Sun X., Wang Y., Li J., Zhang X., Zhang H., Pan Y., Song Z., Yang J., Lu J. (2017). Monolayer Bismuthene-Metal
Contacts:
A Theoretical Study. ACS Appl. Mater. Interfaces.

[14] Lazanas A. C., Tsirka K., Paipetis A. S., Prodromidis M. I. (2020). 2D Bismuthene/Graphene
Modified Electrodes for the Ultra-Sensitive Stripping Voltammetric
Determination of Lead and Cadmium. Electrochim.
Acta.

[15] Cheng X., Li D., Wu Y., Xu R., Yu Y. (2019). Bismuth Nanospheres
Embedded in Three-Dimensional (3D) Porous Graphene Frameworks as High
Performance Anodes for Sodium- and Potassium-Ion Batteries. J. Mater. Chem. A.

[16] Yan L., Gu Z., Zheng X., Zhang C., Li X., Zhao L., Zhao Y. (2017). Elemental Bismuth–Graphene Heterostructures for Photocatalysis
from Ultraviolet to Infrared Light. ACS Catal..

[17] Jiménez
de Castro M., Cabello F., Toudert J., Serna R., Haro-Poniatowski E. (2014). Potential of Bismuth Nanoparticles Embedded in a Glass
Matrix for Spectral-Selective Thermo-Optical Devices. Appl. Phys. Lett..

[18] Inagaki T., Arakawa E. T., Cahters A. R., Glastad K. A. (1982). Optical Properties
of Liquid Pb and Bi between 0.6 and 3.7 eV. Phys. Rev. B.

[19] Elibol K., van Aken P. A. (2022). Uncovering the Evolution of Low-Energy Plasmons in
Nanopatterned Aluminum Plasmonics on Graphene. Nano Lett..

[20] Huang H., Wong S. L., Wang Y., Sun J.-T., Gao X., Wee A. T. S. (2014). Scanning Tunneling Microscope and Photoemission Spectroscopy
Investigations of Bismuth on Epitaxial Graphene on SiC (0001). J. Phys. Chem. C.

[21] Shen K., Hua C., Liang Z., Wang Y., Sun H., Hu J., Zhang H., Li H., Jiang Z., Huang H. (2019). Epitaxial Growth of Free-Standing Bismuth Film on Graphene Embedded
with Nontrivial Properties. ACS Appl. Electron.
Mater..

[22] Hu T., Hui X., Zhang X., Liu X., Ma D., Wei R., Xu K., Ma F. (2018). Nanostructured Bi Grown on Epitaxial Graphene/SiC. J. Phys. Chem. Lett..

[23] Scott S., Kral M., Brown S. A. (2005). Crystallographic
Orientation Transition
and Early Stage Growth Characteristics of Thin Bi Films on HOPG. Surf. Sci..

[24] Scott S. A., Kral M. V., Brown S. A. (2005). Growth of Oriented
Bi Nanorods at
Graphite Step-Edges. Phys. Rev. B.

[25] McCarthy D. N., Robertson D., Kowalczyk P. J., Brown S. A. (2010). The Effects of Annealing
and Growth Temperature on the Morphologies of Bi Nanostructures on
HOPG. Surf. Sci..

[26] Kowalczyk P., Mahapatra O., Le Ster M., Brown S., Bian G., Wang X., Chiang T.-C. (2017). Single Atomic Layer Allotrope of
Bismuth with Rectangular Symmetry. Phys. Rev.
B.

[27] Wang H., Jing J., Henriksen P. (1993). Onset of Crystal
Growth of Bismuth
on Graphite: An Atomic Force Microscopy Study. J. Vac. Sci. Technol. Vac. Surf. Films.

[28] Song F., Wells J. W., Jiang Z., Saxegaard M., Wahlström E. (2015). Low-Temperature Growth of Bismuth
Thin Films with (111)
Facet on Highly Oriented Pyrolytic Graphite. ACS Appl. Mater. Interfaces.

[29] Zayed M. K., Elsayed-Ali H. E. (2005). Condensation on (002) Graphite of Liquid Bismuth Far
below Its Bulk Melting Point. Phys. Rev. B.

[30] Nagao T., Yaginuma S., Saito M., Kogure T., Sadowski J., Ohno T., Hasegawa S., Sakurai T. (2005). Strong Lateral Growth
and Crystallization via Two-Dimensional Allotropic Transformation
of Semi-Metal Bi Film. Surf. Sci..

[31] Bayer B. C., Kaindl R., Reza Ahmadpour
Monazam M., Susi T., Kotakoski J., Gupta T., Eder D., Waldhauser W., Meyer J. C. (2018). Atomic-Scale in Situ Observations of Crystallization
and Restructuring Processes in Two-Dimensional MoS2 Films. ACS Nano.

[32] Elibol K., Mangler C., Gupta T., Zagler G., Eder D., Meyer J. C., Kotakoski J., Bayer B. C. (2020). Process Pathway
Controlled Evolution of Phase and Van-Der-Waals Epitaxy in In/In2O3
on Graphene Heterostructures. Adv. Funct. Mater..

[33] Fuchs D., Bayer B. C., Gupta T., Szabo G. L., Wilhelm R. A., Eder D., Meyer J. C., Steiner S., Gollas B. (2020). Electrochemical
Behavior of Graphene in a Deep Eutectic Solvent. ACS Appl. Mater. Interfaces.

[34] Choi J., Koo S., Song M., Jung D. Y., Choi S.-Y., Ryu S. (2020). Varying Electronic
Coupling at Graphene–Copper Interfaces Probed with Raman Spectroscopy. 2D Mater..

[35] Hofmann P. (2006). The Surfaces
of Bismuth: Structural and Electronic Properties. Prog. Surf. Sci..

[36] Huang P. Y., Ruiz-Vargas C. S., van der Zande A. M., Whitney W. S., Levendorf M. P., Kevek J. W., Garg S., Alden J. S., Hustedt C. J., Zhu Y., Park J., McEuen P. L., Muller D. A. (2011). Grains and Grain
Boundaries in Single-Layer Graphene Atomic Patchwork Quilts. Nature.

[37] Hummel S., Elibol K., Zhang D., Sampathkumar K., Frank O., Eder D., Schwalb C., Kotakoski J., Meyer J. C., Bayer B. C. (2021). Direct Visualization of Local Deformations
in Suspended Few-Layer Graphene Membranes by Coupled in Situ Atomic
Force and Scanning Electron Microscopy. Appl.
Phys. Lett..

[38] Migita S., Kasai Y., Ota H., Sakai S. (1997). Self-Limiting Process
for the Bismuth Content in Molecular Beam Epitaxial Growth of Bi 2
Sr 2 CuO y Thin Films. Appl. Phys. Lett..

[39] Gupta T., Rosza N., Sauer M., Goetz A., Winzely M., Rath J., Naghdi S., Lechner A., Apaydin D. H., Cherevan A., Friedbacher G., Foelske A., Skoff S. M., Bayer B. C., Eder D. (2022). Sonochemical
Synthesis of Large Two-Dimensional
Bi2O2CO3 Nanosheets for Hydrogen Evolution in Photocatalytic Water
Splitting. Adv. Sustain. Syst..

[40] Renucci J. B., Richter W., Cardona M., SchÖstherr E. (1973). Resonance
Raman Scattering in Group Vb Semimetals: As, Sb, and Bi. Phys. Status Solidi B.

[41] Haro-Poniatowski E., Jouanne M., Morhange J. F., Kanehisa M., Serna R., Afonso C. N. (1999). Size Effects Investigated by Raman Spectroscopy in
Bi Nanocrystals. Phys. Rev. B.

[42] Kumari L., Lin J.-H., Ma Y.-R. (2008). Laser Oxidation
and Wide-Band Photoluminescence
of Thermal Evaporated Bismuth Thin Films. J.
Phys. Appl. Phys..

[43] Mitch M. G., Chase S. J., Fortner J., Yu R. Q., Lannin J. S. (1991). Phase Transition
in Ultrathin Bi Films. Phys. Rev. Lett..

[44] Trentelman K. A. (2009). Note on
the Characterization of Bismuth Black by Raman Microspectroscopy. J. Raman Spectrosc..

[45] Aktürk O. Ü., Tomak M. (2010). Bismuth Doping of Graphene. Appl.
Phys. Lett..

[46] Nagao T., Sadowski J. T., Saito M., Yaginuma S., Fujikawa Y., Kogure T., Ohno T., Hasegawa Y., Hasegawa S., Sakurai T. (2004). Nanofilm Allotrope
and Phase Transformation of Ultrathin
Bi Film on Si(111)-7 × 7. Phys. Rev. Lett..

[47] Yimam D. T., Kooi B. J. (2022). Thickness-Dependent
Crystallization of Ultrathin Antimony
Thin Films for Monatomic Multilevel Reflectance and Phase Change Memory
Designs. ACS Appl. Mater. Interfaces.

[48] Peppiatt S. J. (1975). The Melting
of Small Particles. II. Bismuth. Proc. R. Soc.
Math. Phys. Eng. Sci..

[49] Hasegawa M., Watabe M., Hoshino K. (1980). A Theory of Melting in Metallic Small
Particles. J. Phys. F Met. Phys..

[50] Kryshtal A. P., Gladkikh N. T., Sukhov R. V. (2011). Features
of Island Nanostructures
Formed by Melting Sn, Bi and Sn–Bi Thin Films on C Substrates. Appl. Surf. Sci..

[51] Olson E. A., Efremov M. Yu., Zhang M., Zhang Z., Allen L. H. (2005). Size-Dependent
Melting of Bi Nanoparticles. J. Appl. Phys..

[52] Li Y., Zang L., Jacobs D. L., Zhao J., Yue X., Wang C. (2017). In Situ Study on Atomic
Mechanism of Melting and Freezing of Single
Bismuth Nanoparticles. Nat. Commun..

[53] Li J., Chen J., Wang H., Chen N., Wang Z., Guo L., Deepak F. L. (2018). In Situ
Atomic-Scale Study of Particle-Mediated Nucleation
and Growth in Amorphous Bismuth to Nanocrystal Phase Transformation. Adv. Sci..

[54] Lee J., Yang J., Kwon S. G., Hyeon T. (2016). Nonclassical Nucleation
and Growth of Inorganic Nanoparticles. Nat.
Rev. Mater..

[55] Li J., Wang Z., Deepak F. L. (2018). Direct
Atomic-Scale Observation of
Intermediate Pathways of Melting and Crystallization in Supported
Bi Nanoparticles. J. Phys. Chem. Lett..

[56] Jenc̆ic̆ I., Bench M. W., Robertson I. M., Kirk M. A. (1995). Electron-beam-induced
Crystallization of Isolated Amorphous Regions in Si, Ge, GaP, and
GaAs. J. Appl. Phys..

[57] Wang L., Wang Q., Xian A., Lu K. (2003). Precise Measurement
of the Densities of Liquid Bi, Sn, Pb and Sb. J. Phys.: Condens. Matter.

[58] Gusmão R., Sofer Z., Bouša D., Pumera M. (2017). Pnictogen (As, Sb,
Bi) Nanosheets for Electrochemical Applications Are Produced by Shear
Exfoliation Using Kitchen Blenders. Angew. Chem.,
Int. Ed..

[59] Ren Y., Zhang Z., Lu Y., Wu N., Tang Y., Yu Y., Wang H.-T. (2025). Multilayer
A17 Black Antimonene via van Der Waals Epitaxy. ACS Nano.

[60] Kryshtal A., Bogatyrenko S., Khshanovska O. (2023). Direct Imaging of Surface Melting
on a Single Sn Nanoparticle. Nano Lett..

[61] Jiang N., Su D., Spence J. C., Zhou S., Qiu J. (2009). Volume Plasmon of Bismuth
Nanoparticles. Solid State Commun..

[62] Powell C. (1960). The Origin
of the Characteristic Electron Energy Losses in Ten Elements. Proc. Phys. Soc..

[63] Borja-Urby R., Paredes-Carrera S. P., Viltres-Cobas H., Santiago-Jacinto P., Paraguay-Delgado F., Herrera-Pérez G., Rendón-Vázquez L., Sánchez-Ochoa J. C., Morales-Cruz D. (2019). Confined Volume
Plasmon Spatial Distribution by Low-Loss EELS on Self-Assemble Bismuth
Nanoparticles. J. Electron Spectrosc. Relat.
Phenom..

[64] Wang Y. W., Kim J. S., Kim G. H., Kim K. S. (2006). Quantum Size Effects
in the Volume Plasmon Excitation of Bismuth Nanoparticles Investigated
by Electron Energy Loss Spectroscopy. Appl.
Phys. Lett..

[65] Wang Y., Kim J.-S., Lee J. Y., Kim G. H., Kim K. S. (2007). Diameter-
and Length-Dependent Volume Plasmon Excitation of Bismuth Nanorods
Investigated by Electron Energy Loss Spectroscopy. Chem. Mater..

[66] Peng S., McMahon J. M., Schatz G. C., Gray S. K., Sun Y. (2010). Reversing
the Size-Dependence of Surface Plasmon Resonances. Proc. Natl. Acad. Sci. U. S. A..

[67] Polking M. J., Jain P. K., Bekenstein Y., Banin U., Millo O., Ramesh R., Alivisatos A. P. (2013). Controlling
Localized Surface Plasmon
Resonances in GeTe Nanoparticles Using an Amorphous-to-Crystalline
Phase Transition. Phys. Rev. Lett..

[68] Tian Y., Toudert J. (2018). Nanobismuth: Fabrication,
Optical, and Plasmonic Properties—Emerging
Applications. J. Nanotechnol.

[69] Haro-Poniatowski E., Serna R., Suárez-García A., Afonso C. N. (2005). Thermally Driven Optical Switching in Bi Nanostructures. Nanotechnology.

[70] Toudert J., Serna R., Jiménez de Castro M. (2012). Exploring
the Optical
Potential of Nano-Bismuth: Tunable Surface Plasmon Resonances in the
Near Ultraviolet-to-Near Infrared Range. J.
Phys. Chem. C.

