# Supplementary material for: Structural and Plasmonic Evolution in Mixed-Dimensionality Bismuth/Graphene Heterostructures
Source: ACS Appl Mater Interfaces. 2026 Mar 3;18(10):15430–43. doi: 10.1021/acsami.5c20752 (PMC13006955; doi:10.1021/acsami.5c20752)
Supplement: Supplementary file 1 [file am5c20752_si_001.pdf]

Supporting Information to:

Structural and Plasmonic Evolution in Mixed-Dimensionality Bismuth/Graphene Heterostructures

Tushar Gupta,[1,*] Kenan Elibol,[2,3] Michael Stöger-Pollach,[4] Kimmo Mustonen,[2] Clemens Mangler,[2] Jannik C. Meyer,[2,5] Jani Kotakoski,[2] Bernhard C. Bayer,[1,2,*] Dominik Eder[1]

1. Institute of Materials Chemistry, Technische Universität Wien (TU Wien), Getreidemarkt 9/165, A-1060 Vienna, Austria

2. University of Vienna, Faculty of Physics, Boltzmanngasse 5, A-1090 Vienna, Austria

3. Max Planck Institute for Solid State Research, Heisenbergstrasse 1, 70569 Stuttgart, Germany

4. USTEM, Technische Universität Wien (TU Wien), Wiedner Hauptstrasse 8-10, A-1040 Vienna, Austria

5. Institute of Applied Physics, Eberhard Karls University of Tuebingen, Auf der Morgenstelle 10, D-72076 Tuebingen, Germany

*Corresponding authors: bernhard.bayer-skoff@tuwien.ac.at (B.C.B), tusgu@dtu.dk (T.G.)

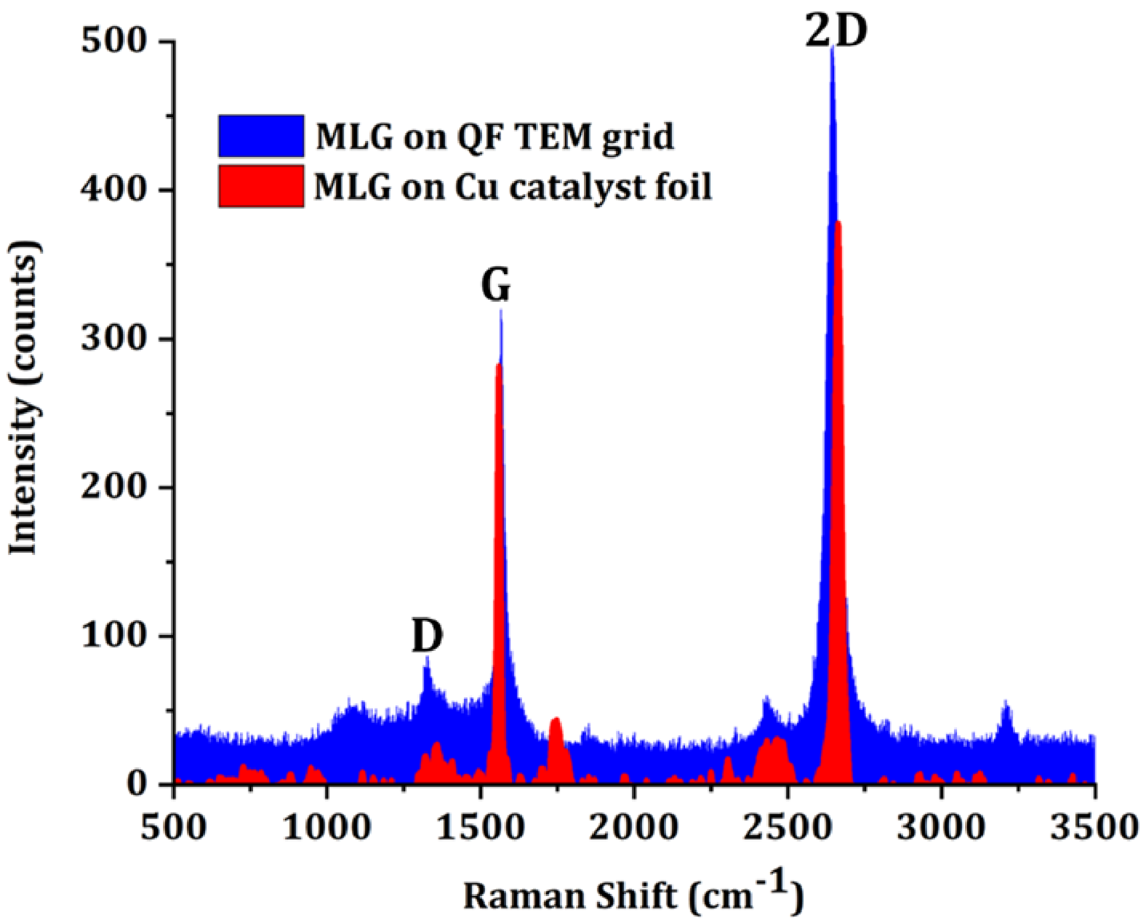

Figure S1. Raman spectra of the high-quality, CVD monolayer graphene (MLG) used for Bi deposition in suspended form on TEM grid[1] (blue, commercial Graphenea CVD monolayer graphene-covered Quantifoil (QF) TEM grid) and in supported form on CVD Cu catalyst foil (red), both showing a 2D/G intensity ratio and low D peak, consistent with high-quality monolayer CVD graphene.[2–5] These measurements employed a WITec alpha 300 RSA+ system with laser wavelength of 488 nm, which reduces photoluminescence background and results in less peak broadening and intensity changes from Cu support compared to the 532 nm excitation used in Fig. 1d.[4]

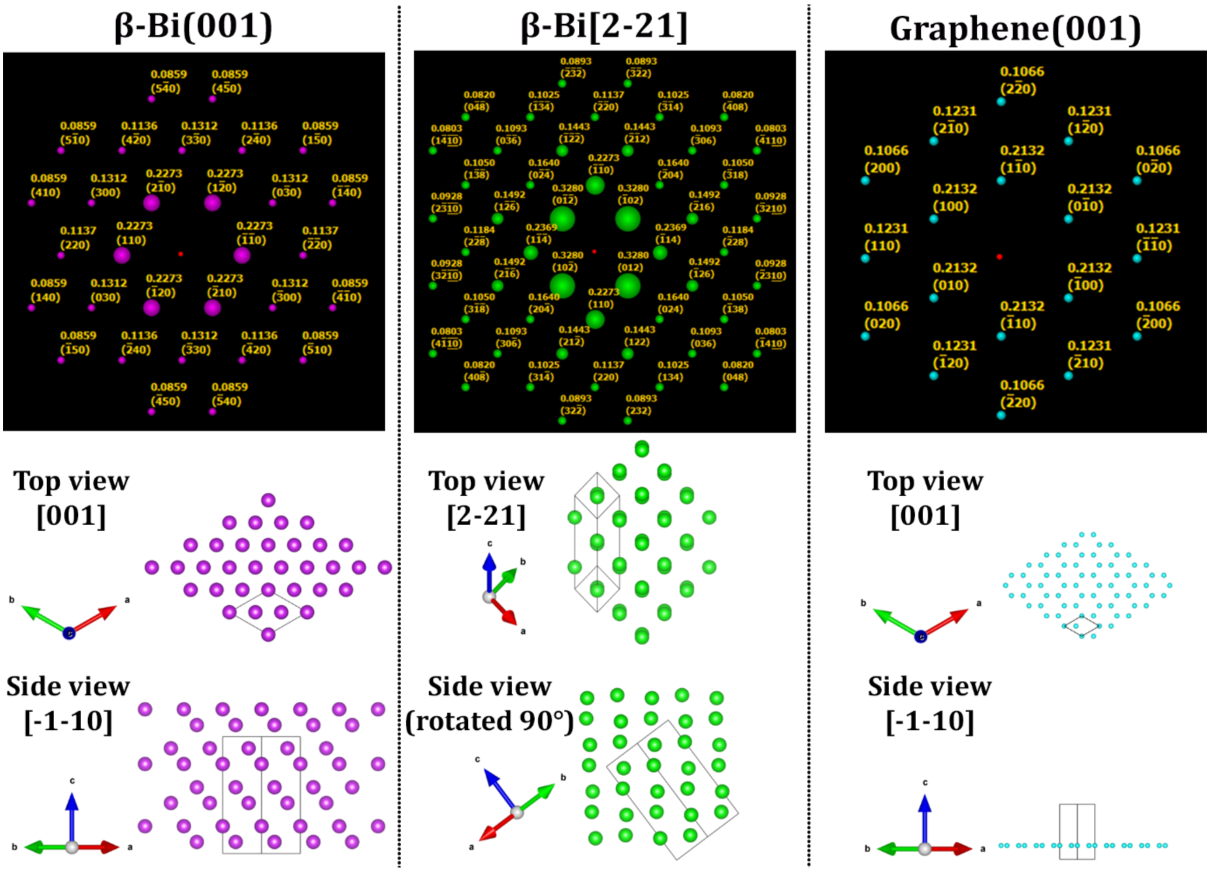

Figure S2. Atomic models and simulated electron diffraction patterns. Simulated Fourier Transform (FT) / Selected area electron diffraction (SAED) patterns (top) and corresponding atomic models in top/plan view (middle) and side view (below) of β-Bi(001), β-Bi[2-21] and graphene(001) respectively. The models in top view correspond to the atomic structure in Annular dark field (ADF) scanning transmission electron microscope ((S)TEM) and bright field transmission electron microscope (BF TEM) images of the Bismuth (Bi) deposits on graphene. Unit cells and axes are also plotted and the zone axis for all views is indicated. Note that FT/SAED can have an arbitrary rotational offset with respect to the atomic top view sketches. Following structure files were predominantly used: β-Bi (A7, rhombohedral, R-3m): 04-007-5315/53796[6] (from the Pdf 4+ crystallographic database/ Inorganic Crystal Structure Database ICSD collection code) and for graphene (0-056-0159[7]). Structure visualization was undertaken via Vesta software.[8]

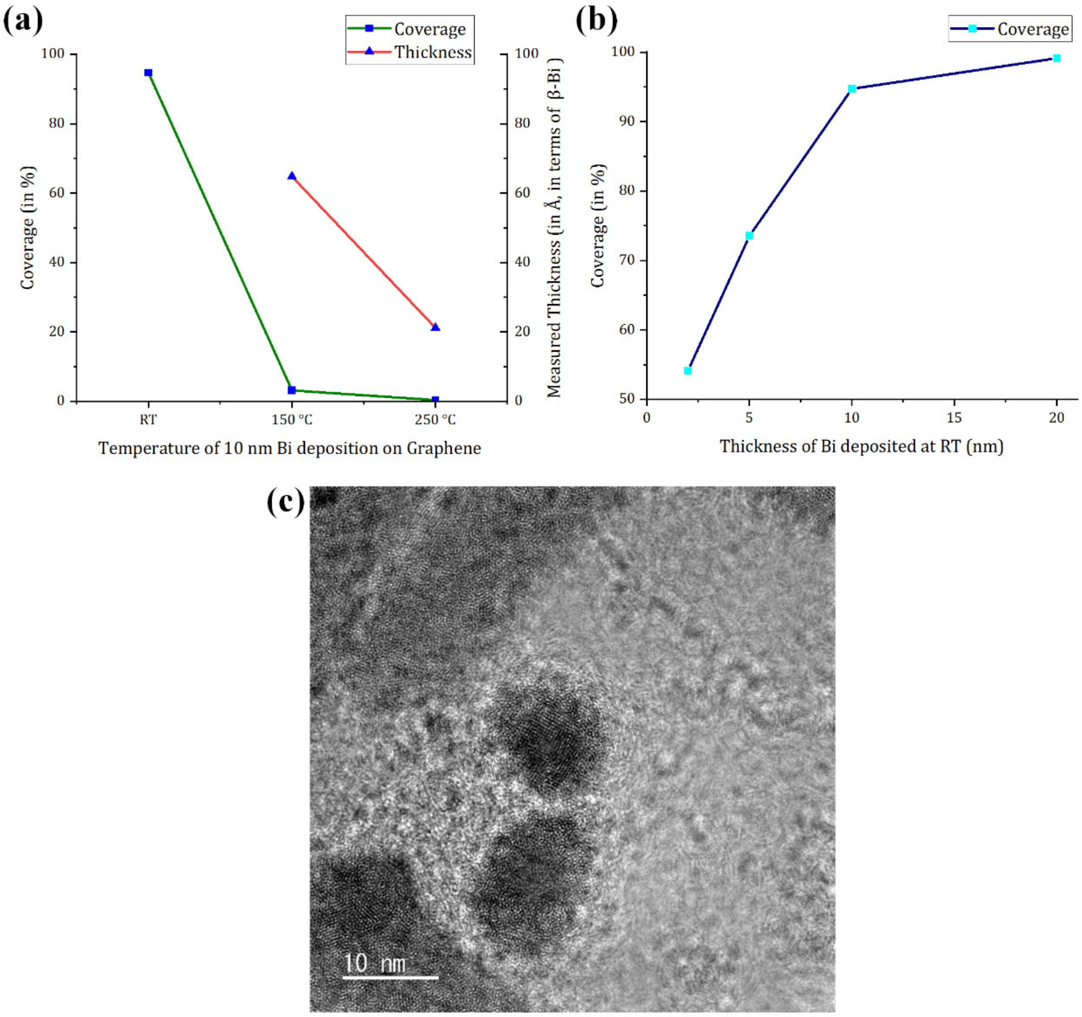

Figure S3. (a) The percentage areal coverage of graphene support underneath by 10 nm Bi deposits at different temperatures, namely: RT, 150 ℃ and 250 ℃ (shown by green interconnecting line with blue squares) for the BF TEM images shown in Fig. 1. The thickness of the Bi deposits analysed via intensity analysis of ADF (S)TEM images (using atomically-clean monolayer graphene regions as an intensity reference for the β-Bi of known structure but of unknown thickness via intensity dependence on atomic number $Z^{\sim 1.6}$ relation[9–11]) for nominally 10 nm Bi deposits (that are widely spaced on graphene) from different temperatures, namely: 150 ℃ and 250 ℃ (shown by red interconnecting line with blue triangles). (b) The percentage areal coverage of graphene underneath by RT deposition of Bi of different thicknesses, namely: 2 nm, 5 nm, 10 nm and 20 nm for the BF TEM images shown in Fig. 4.

(c) Higher magnification BF TEM image of the small, darker contrast particles in Fig. 4a (2 nm, RT deposition), showing that these are small crystals with β-Bi[2-21] structure.

10 nm Bi @ 150 °C

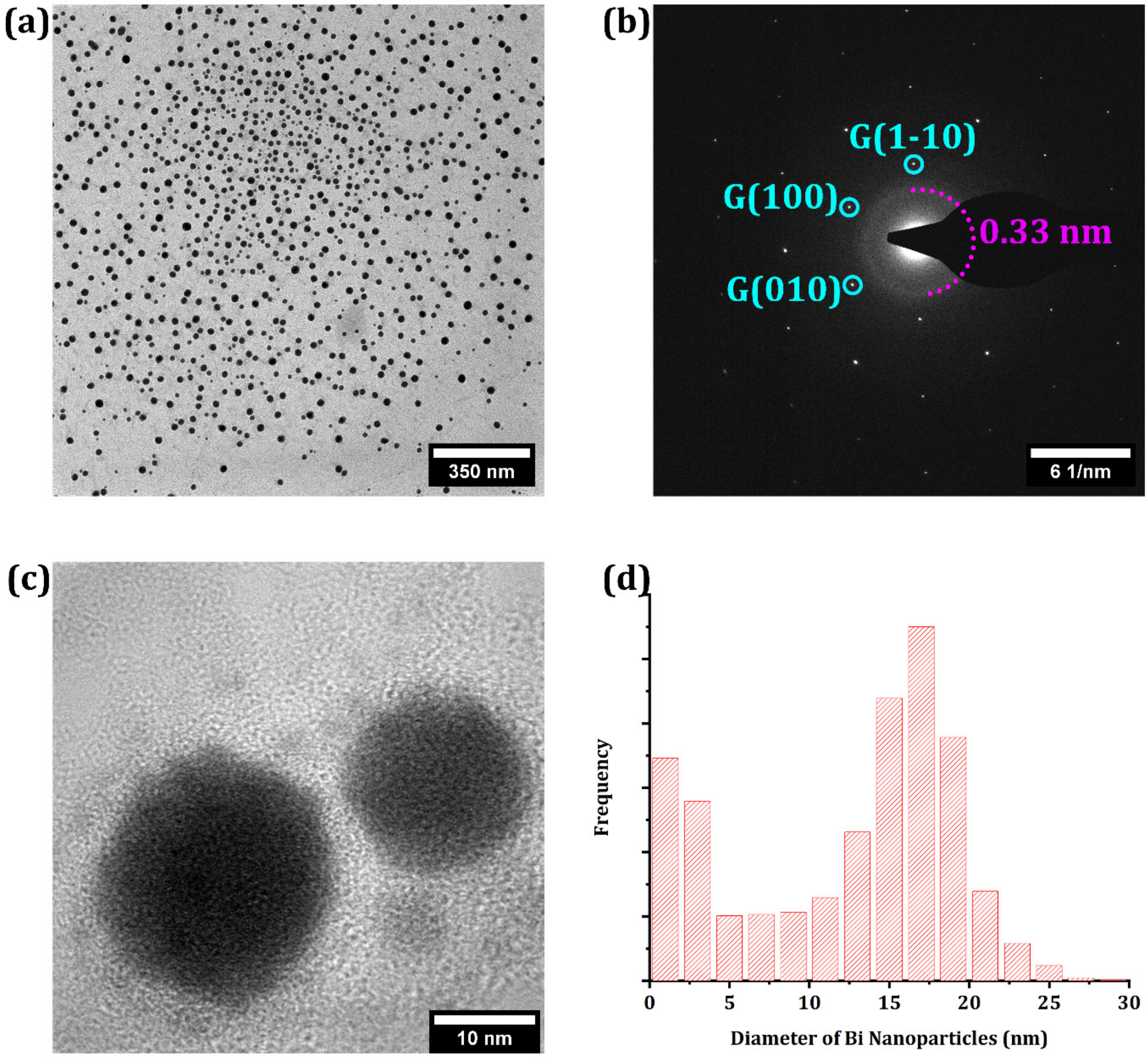

Figure S4. 10 nm Bi deposits at 150 °C. BF-TEM image (a) shows a BF TEM image of the 10 nm Bi deposits at 150 °C on freely suspended graphene support. (b) SAED on (a) shows the pristine quality of the single crystalline monolayer graphene underneath utilized as substrate for Bi growth. Graphene spots have been marked with cyan colour. The faint amorphous ring at d spacing of 0.33 nm confirms the amorphous nature of the Bi deposits as 0.33 nm (shown with magenta colour) corresponds to β-Bi[2-21] as shown in Supporting Fig. S2c. (c) is the BF-TEM image of two of the NPs in (a) showing the amorphous nature of the 10 nm Bi deposits at 150 °C. (d) shows the size distribution of the Bi NPs for 10 nm Bi deposits at 150 °C. The NPs were found to have an average diameter of around 17 nm.

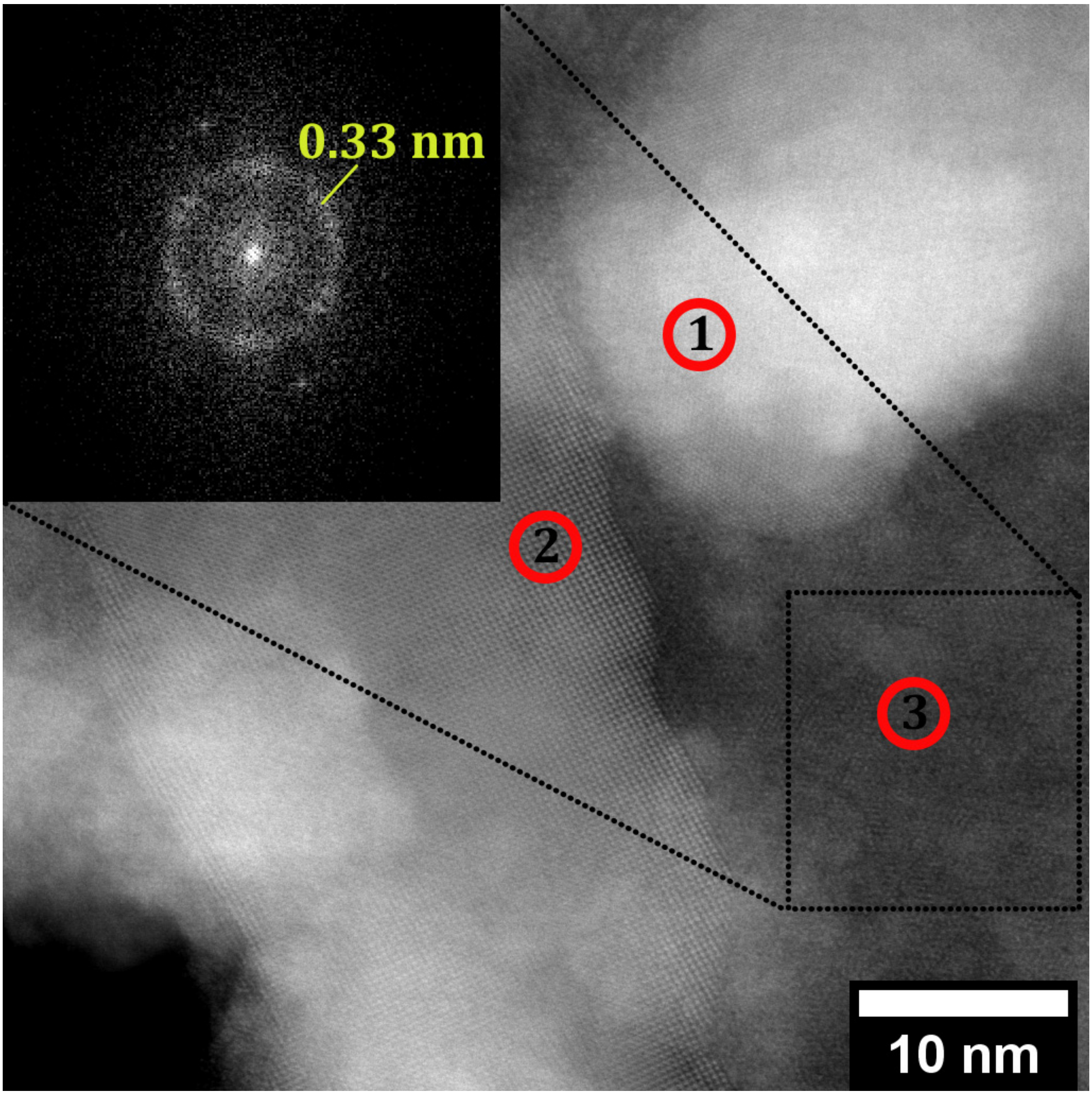

Figure S5. Region 3 in Fig. 2. ADF (S)TEM images of 10 nm Bi films on graphene at RT outlining region 3 (as indexed in Fig. 2a,b) i.e. the amorphous β-Bi[2-21]. The FT pattern in the inset is recorded on the region 3 (marked with black dotted square). The ring in the FT pattern at d-spacing of 0.33 nm indicates the preferred orientation of this amorphous Bi phase to β-Bi[2-21].

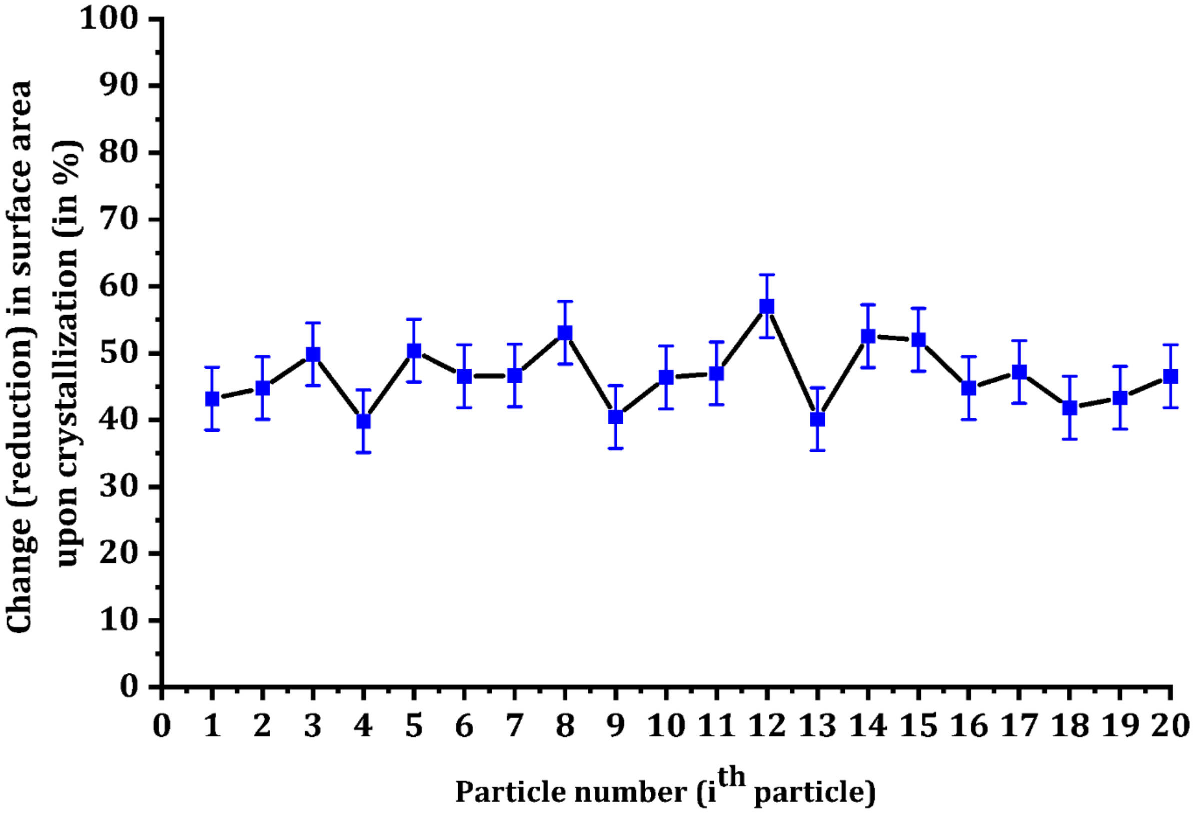

Figure S6. Change in surface area of the amorphous NP upon crystallization. The plot shows the % reduction in the surface area of the NPs in top view as per example in the BF TEM images in Fig. 5, 6 and similar data from their initial amorphous state to final crystallized state. The plot shows the change in area calculated for 20 such NPs for 10 nm Bi depositions at 150 °C and 250 °C. On an average, a reduction of 45 % of top surface area was observed in the initial amorphous NPs upon their complete ordered restructuring.

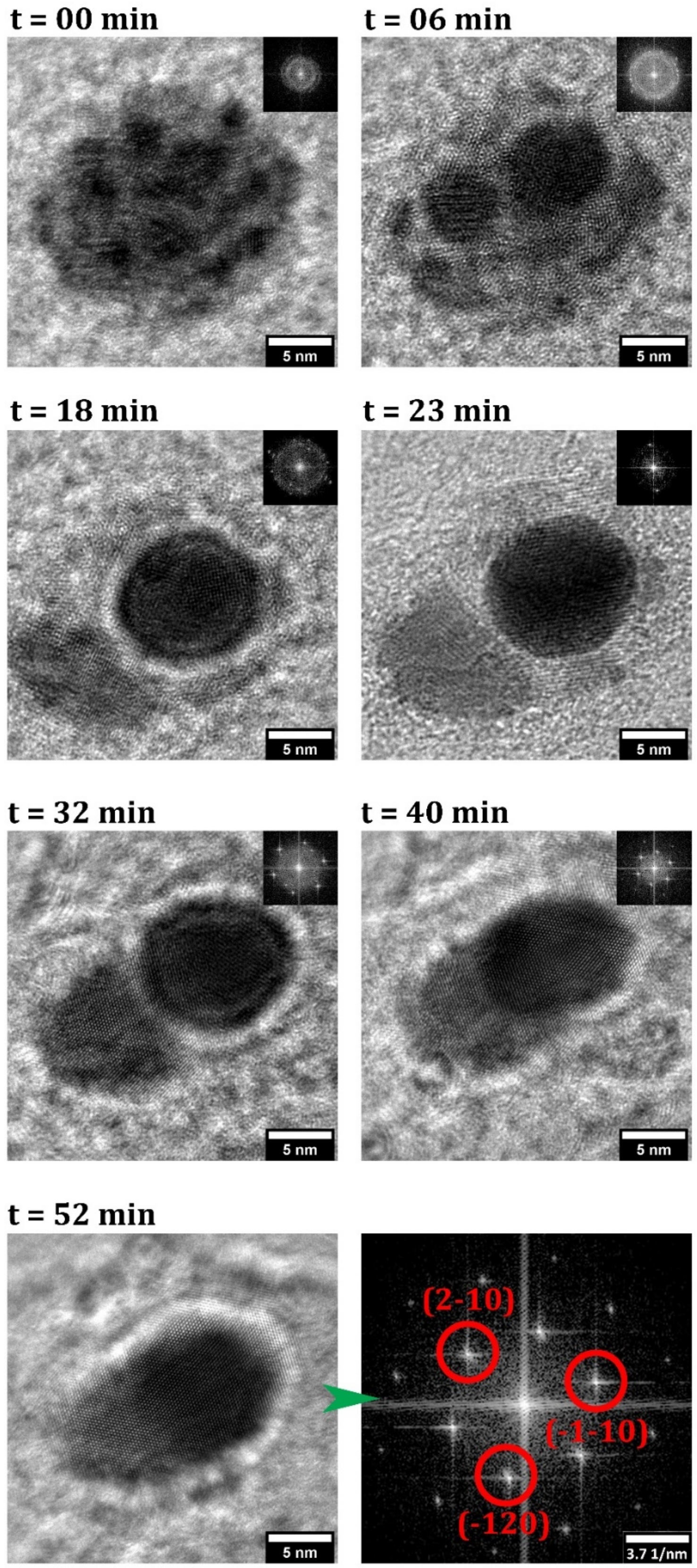

Figure S7. Coalescence and restructuring of two isolated NPs to β-Bi(001). ADF BF TEM image of the electron beam induced crystallization of Bi NPs (from 10 nm Bi deposited on

graphene at 150 °C) where two isolated NPs were observed merging to β-Bi(001) crystallized NP suggesting the higher stability of β-Bi(001) with increasing thickness.

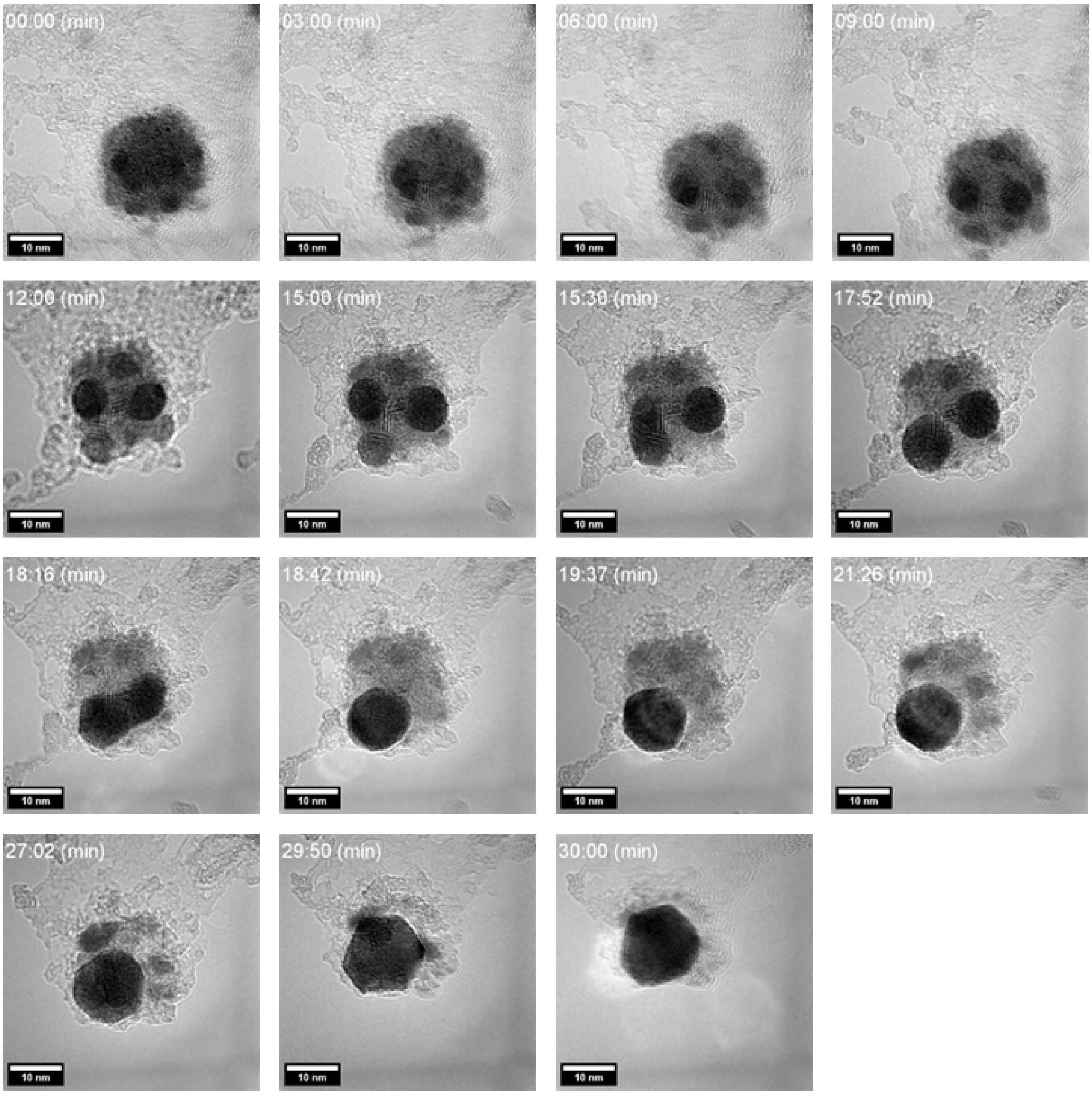

Figure S8. Crystallization of Bi nanoparticles under electron beam at high temporal resolution. The TEM micrographs at different time stamps captured at higher temporal resolution. The TEM micrographs presented here are adopted from Supporting Video 1.

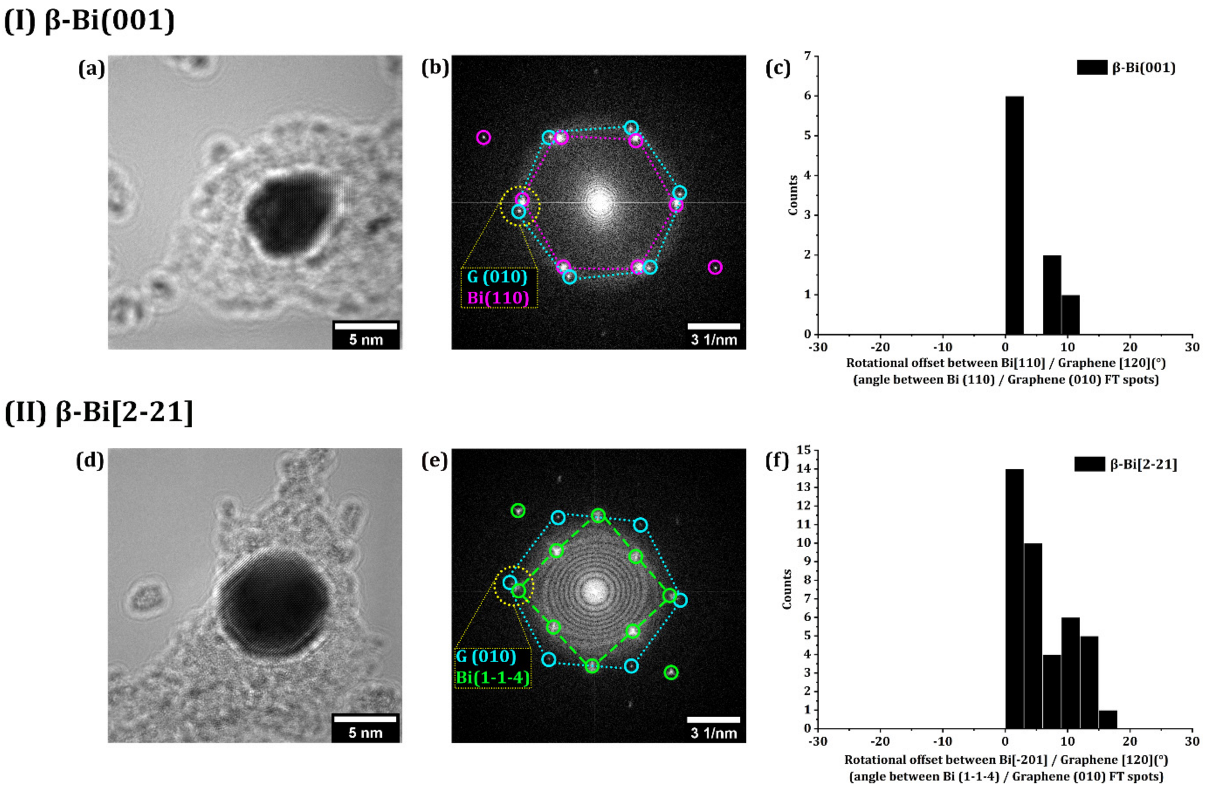

Figure S9. Epitaxial orientation statistics. BF-TEM image (a) shows the electron beam crystallized β-Bi(001) particle (10 nm Bi at 150 °C) and the graphene support with corresponding FT pattern (b) from the BF-image showing β-Bi(001) (magenta) and graphene(001) (cyan) reflections indicating signs of the following rotational vdW epitaxy relation of β-Bi(001) with respect to graphene(001) underneath: β-Bi(001)||graphene(001) / β-Bi[110]||graphene[120], similar to what was observed for RT deposits in Fig. 3. BF-TEM image (d) shows the electron beam crystallized β-Bi[2-21] particle (10 nm Bi at 150 °C) and the graphene support with corresponding FT pattern (e) from the BF-image showing β-Bi[2-21] (green) and graphene(001) (cyan) reflections indicating signs of the following rotational vdW epitaxy relation of β-Bi[2-21] with respect to graphene(001) underneath: β-Bi[2-21]⊥graphene(001)/β-Bi[-201]||graphene[120], similar to what was observed for RT deposits in Fig. 3. (c) and (f) show histogram of multiple measurements of misorientation angles between Bi[110] and graphene[120] in the crystallized β-Bi(001) and β-Bi[2-21] respectively

with respect to graphene underneath, showing that most prevalent misorientations correspond to the cases in (a,b) and (d,e), in line with RT results in Fig. 3.

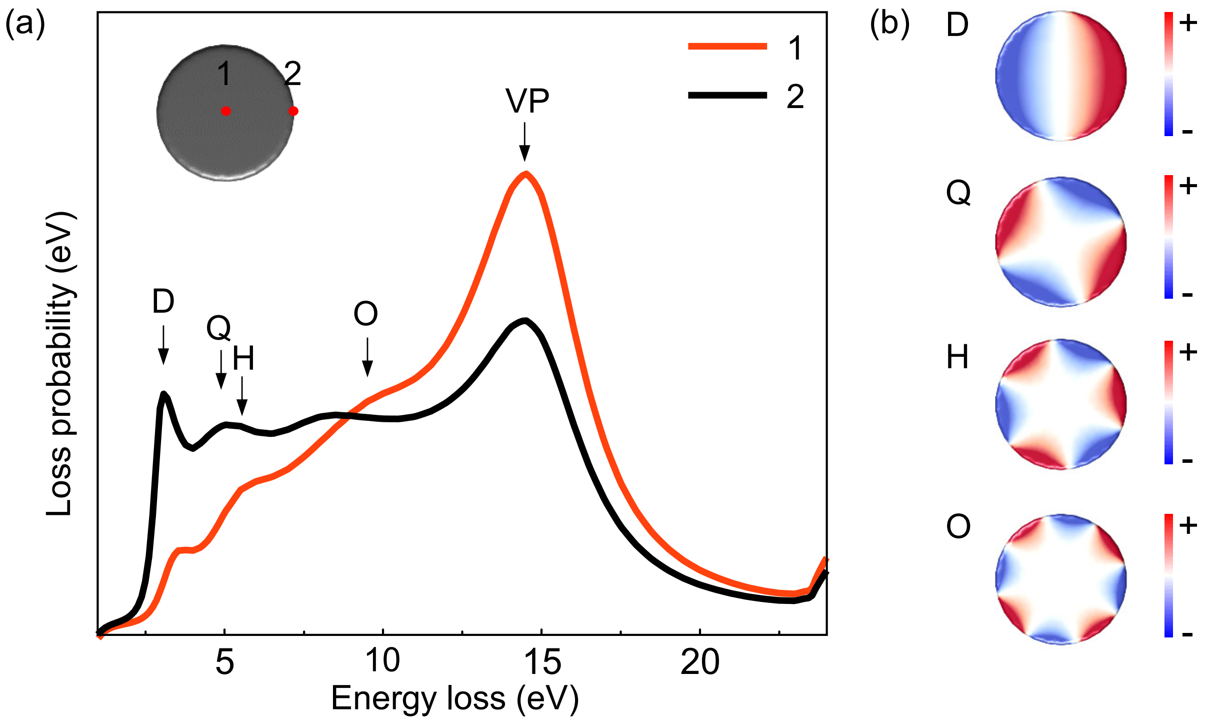

Figure S10. SPs and VP in Bi NPs. (a) To accompany our experimental EELS data[12,13] in Fig. 7, we here present simulated EEL spectra of a Bi NP with a lateral size of 50 nm and a thickness of 10 nm reveal a VP at 14.5 eV, which is in good agreement with experimental data obtained from a Bi NP of comparable size.[14] Note that our experimentally observed VP in Fig. 7 is at ~19 eV for smaller ~11 nm to 13 nm particles, which is consistent with prior reports of particles of similar size,[14] see main text discussion. It should be noted that our classical boundary element method (BEM) simulations do not account for quantum confinement effects. Therefore, prior experimentally observed size-related (VP) energy shifts[14] are not reproduced in our simulated spectra. (b) Surface eigencharge distributions showing the SP modes identified from the EEL spectra in (a). The observed peaks in (a) can be tentatively attributed to possibly localized SP modes corresponding to the dipole (D), quadrupole (Q), hexapole (H) and octupole (O) excitations. Simulations were performed as following: Boundary element method (BEM) simulations of EEL spectra were performed using the MNPBEM toolbox in MATLAB.[15] The simulations employed an electron beam excitation with an energy of 60 keV. The dielectric function of Bi was taken from the work of Hagemann et al..[16]

Table S1. Sb vs. Bi PVD space on graphene. Comparison of findings in Sb/graphene and Bi/graphene systems under comparable synthesis and processing conditions from PVD route, as in this work and our prior work.[17]

**Parameters**	**10 nm Sb on graphene (via PVD)**	**10 nm Bi on graphene (via PVD)**
**RT Deposition**	Fully closed amorphous films	Fully closed largely crystalline films
**Deposition at 150 °C and 250 °C**	Crystalline nanostructures with lower areal coverage	Amorphous spherical nanoparticles with lower areal coverage
**Structure (if crystalline)**	At 150 °C and 250 °C:<br>β-Sb(001).<br>β-Sb[2-21]/cubic-Sb(001).	At RT:<br>Thick deposits = β-Bi(001).<br>Thin deposits = β-Bi[2-21]<br>Thinner deposits = nearing crystallization amorphous β-Bi[2-21]-like.
**Oxidation susceptibility**	Presence of superficial surface Sb-oxide in Sb structures after a period of 8 months.	No such discrete signs of any type of oxidation were found
**Electron beam dynamics**	All Sb deposits (at RT, 150 °C and 250 °C) = static under electron beam	RT crystalline Bi films = static under electron beam<br>Amorphous Bi nanoparticles (at 150 °C and 250 °C) = electron beam induced crystallization, along with associated surface plasmon shift

Supporting References

(1) Regan, W.; Alem, N.; Alemán, B.; Geng, B.; Girit, Ç.; Maserati, L.; Wang, F.; Crommie, M.; Zettl, A. A Direct Transfer of Layer-Area Graphene. *Appl. Phys. Lett.* **2010**, *96*, 113102.

(2) Fuchs, D.; Bayer, B. C.; Gupta, T.; Szabo, G. L.; Wilhelm, R. A.; Eder, D.; Meyer, J. C.; Steiner, S.; Gollas, B. Electrochemical Behavior of Graphene in a Deep Eutectic Solvent. *ACS Appl. Mater. Interfaces* **2020**, *12*, 40937–40948.

(3) Fickl, B.; Seifried, T. M.; Rait, E.; Genser, J.; Wicht, T.; Kotakoski, J.; Rupprechter, G.; Lugstein, A.; Zhang, D.; Dipolt, C.; Grothe, H.; Eder, D.; Bayer, B. C. Controllable Freezing Transparency for Water Ice on Scalable Graphene Films on Copper. *ArXiv Prepr. ArXiv240315629* **2024**.

(4) Choi, J.; Koo, S.; Song, M.; Jung, D. Y.; Choi, S.-Y.; Ryu, S. Varying Electronic Coupling at Graphene–Copper Interfaces Probed with Raman Spectroscopy. *2D Mater.* **2020**, *7*, 025006.

(5) Kidambi, P. R.; Bayer, B. C.; Blume, R.; Wang, Z.-J.; Baehtz, C.; Weatherup, R. S.; Willinger, M.-G.; Schloegl, R.; Hofmann, S. Observing Graphene Grow: Catalyst–Graphene Interactions during Scalable Graphene Growth on Polycrystalline Copper. *Nano Lett.* **2013**, *13*, 4769–4778.

(6) Cucka, P.; Barrett, C. S. The Crystal Structure of Bi and of Solid Solutions of Pb, Sn, Sb and Te in Bi. *Acta Crystallogr.* **1962**, *15*, 865–872.

(7) Howe, J. Y.; Rawn, C. J.; Jones, L. E.; Ow, H. Improved Crystallographic Data for Graphite. *Powder Diffr.* **2003**, *18*, 150–154.

(8) Momma, K.; Izumi, F. VESTA: A Three-Dimensional Visualization System for Electronic and Structural Analysis. *J. Appl. Crystallogr.* **2008**, *41*, 653–658.

(9) Krivanek, O. L.; Chisholm, M. F.; Nicolosi, V.; Pennycook, T. J.; Corbin, G. J.; Dellby, N.; Murfitt, M. F.; Own, C. S.; Szilagyi, Z. S.; Oxley, M. P.; Pantelides, S. T.; Pennycook, S. J. Atom-by-Atom Structural and Chemical Analysis by Annular Dark-Field Electron Microscopy. *Nature* **2010**, *464*, 571–574.

(10) Niggas, A.; Schwestka, J.; Creutzburg, S.; Gupta, T.; Eder, D.; Bayer, B.; Aumayr, F.; Wilhelm, R. The Role of Contaminations in Ion Beam Spectroscopy with Freestanding 2D Materials: A Study on Thermal Treatment. *J. Chem. Phys.* **2020**, *153*, 014702.

(11) Niggas, A.; Creutzburg, S.; Schwestka, J.; Wöckinger, B.; Gupta, T.; Grande, P. L.; Eder, D.; Marques, J. P.; Bayer, B. C.; Aumayr, F.; Bennett, R.; Wilhelm, R. A. Peeling Graphite Layer by Layer Reveals the Charge Exchange Dynamics of Ions inside a Solid. *Commun. Phys.* **2021**, *4*, 180.

(12) Colliex, C. Electron Energy-Loss Spectroscopy (EELS) on Nano-Dimensional Structures. *J. Electron Microsc. (Tokyo)* **1999**, *48*, 995–1003.

(13) Trasobares, S.; Stéphan, O.; Colliex, C. Time Resolved Electron Energy Loss Spectroscopy as a Tool for Controlling and Monitoring the Early Stages of Electron Beam Induced Transformations. *Microsc. Microanal.* **2002**, *8*, 1384–1385.

(14) Wang, Y. W.; Kim, J. S.; Kim, G. H.; Kim, K. S. Quantum Size Effects in the Volume Plasmon Excitation of Bismuth Nanoparticles Investigated by Electron Energy Loss Spectroscopy. *Appl. Phys. Lett.* **2006**, *88*, 143106.

(15) Hohenester, U. Simulating Electron Energy Loss Spectroscopy with the MNPBEM Toolbox. *Comput. Phys. Commun.* **2014**, *185*, 1177–1187.

(16) Hagemann, H.-J.; Gudat, W.; Kunz, C. Optical Constants from the Far Infrared to the X-Ray Region: Mg, Al, Cu, Ag, Au, Bi, C, and Al2O3. *J. Opt. Soc. Am.* **1975**, *65*, 742–744.

(17) Gupta, T.; Elibol, K.; Hummel, S.; Stöger-Pollach, M.; Mangler, C.; Habler, G.; Meyer, J. C.; Eder, D.; Bayer, B. C. Resolving Few-Layer Antimonene/Graphene Heterostructures. *Npj 2D Mater. Appl.* **2021**, *5*, 53.
